# Influenza Virus-Induced Paracrine Cellular Senescence of the Lung Contributes to Enhanced Viral Load

**DOI:** 10.14336/AD.2023.0310

**Published:** 2023-08-01

**Authors:** Luise Schulz, Franziska Hornung, Antje Häder, Lukáš Radosa, Axel A Brakhage, Bettina Löffler, Stefanie Deinhardt-Emmer

**Affiliations:** ^1^Institute of Medical Microbiology, Jena University Hospital, Jena, Germany.; ^2^Department of Molecular and Applied Microbiology, Leibniz Institute for Natural Product Research and Infection Biology - Hans Knöll Institute (HKI), Jena, Germany.; ^3^Leibniz Centre for Photonics in Infection Research (LPI), Jena, Germany.; ^4^Else Kröner Graduate School for Medical Students "JSAM", Jena University Hospital, Jena, Germany.

**Keywords:** cellular senescence, influenza A virus, aging, TNF-α, premature senescence

## Abstract

Aging is a major risk factor associated with increased morbidity and mortality rates observed during respiratory infections. In this study, we investigated the role of influenza virus infections in the establishment of premature cellular senescence and paracrine macrophage-activated inflammation. We observed in our murine model a premature aging by the appearance of senescent cells in the lungs after 21 d of influenza A virus infection. By using murine *ex vivo* lung models, the influence of TNF-α on the establishment of cellular senescence was detectable. Our findings were proven by using conditioned media of infected human monocyte-derived macrophages on primary lung fibroblasts. Here, a distinct expression of senescence-associated parameters could be confirmed. Furthermore, senescent cells in the lungs strongly influenced subsequent viral infections. Our data demonstrated a higher viral load in senescent primary lung fibroblasts, indicating an intracellular effect on viral replication. Transcriptomic data revealed an increased regulation of JAK/STAT signaling in senescent IAV-infected cells accompanied with increased TRAIL expression. Additionally, senescent cells indicating low pH values, accelerating viral replication. Our study provides new insights into pathomechanisms of virus-induced cellular senescence. Hence, IAV infection induces premature senescence and subsequent infections in senescent cells lead to an increased viral replication.

## INTRODUCTION

Influenza virus infections are a major source of morbidity and mortality globally (https//www.who.int/news-room/fact-sheets/detail/influenza-(seasonal)). Increased susceptibility to influenza virus infections is a result of chronic diseases, diabetes, obesity, pregnancy, and age [1, 2]. Older adults, defined as those aged 65 years or above, are disproportionately affected by influenza-related morbidity and mortality [3].

Aging is characterized by various degenerative mechanisms that lead to the loss of physiological integrity. Biochemical changes during aging, such as genomic instability, and cellular senescence, have been summarized as hallmarks of aging [4]. Cellular senescence is defined as irreversible cell cycle arrest with some general characteristics such as enlargement, flattening, shape irregularity, and increase in the number of vacuoles [5, 6]. These changes result in the secretion of various pro-inflammatory cytokines, chemokines, growth factors, and proteases; called the senescence-associated secretory phenotype (SASP) [7]. SASP factors can be grouped into autocrine (cell-autonomous) and paracrine (non-cell-autonomous) effects. Cell-autonomous SASP factors, such as interleukin (IL)-1α and IL-6, reinforce the senescent state. Non-cell-autonomous factors influence the cellular environment, including the manifestation of senescence in healthy proliferative cells. This form of non-cell-autonomous stable growth arrest is called paracrine senescence [8].

Cell cycle arrest in senescence is mainly mediated via the following major tumor suppressor pathways: the p53/cyclin-dependent kinase inhibitor 1A (p21/WAF1/ CIP1) pathway and cyclin-dependent kinase inhibitor 2A (p16/INK4A)/ retinoblastoma protein (pRB) pathway, and can be induced by cellular stress, leading to DNA damage [6]. In recent years, various mechanisms that induce stable senescence have been elucidated [9].

Moreover, cellular senescence can be prematurely induced by viral infections, such as measles virus, human respiratory syncytial virus and coronavirus, due to cell- and/or non-cell-autonomous mechanisms [10]. Virus-induced senescence cannot be distinguished from the other types of senescence; however, senescence markers and SASP factors have been found in tissue samples of the nasopharyngeal cavity and lungs of patients suffering from coronavirus disease 2019 (COVID-19) with severe disease progression [11].

Influenza A virus (IAV) is leading to an alveolar infection with strong inflammatory response triggered by virus replication and innate immune cells [12]. Alveolar macrophages contribute to immune response by phagocytosis, the secretion of cytokines and chemokines [13]. Here, Tumor Necrosis Factor (TNF)-α is responsible for pleiotropic effects within the alveoli [14].

In this study, we aimed to investigate the impact of IAV infection on (I) the premature induction of cellular senescence via (II) the paracrine effect of TNF-α by using in vivo and in vitro models. In subsequent virus infections, we demonstrate (III) an enhanced viral load in senescent lung cells by using an ex vivo model.

Our data provide new insights into the mechanisms between aging and lung infection and suggest a crucial involvement of viruses in the induction of cellular senescence in the human lung.

## MATERIALS AND METHODS

### Cells and Viruses

Primary human lung fibroblasts (IMR-90s, Coriell Institute for Medical Research, Camden, USA) were cultured in Dulbecco’s modified Eagle’s medium (DMEM, Sigma Aldrich, Taufkirchen, Germany) with 10 % fetal bovine serum (FBS, PAN Biotech, Aidenbach, Germany), and 1 % penicillin/streptomycin (P/S, Lonza, Basel, Schweiz).

Human monocyte-derived macrophages (hMDM) were isolated from healthy donors by using Histopaque (Sigma Aldrich) and separated by density gradient centrifugation, plated in monocyte attachment medium (Promocell, Heidelberg, Germany) for 2 h and cultivated in M1-Macrophage Generation Medium DXF (Promocell) for 7 d.

Madin-Darby canine kidney (MDCK) cells were cultured in Eagle minimum essential medium (EMEM, Sigma Aldrich) with 10 % FBS and 1 % P/S.

In vitro infections were performed using influenza A virus/IAV/H1N1/Puerto Rico/8/1934 (PR8). For the in vivo model the mouse-adapted strain influenza A virus/IAV/H1N1/vi0084/2016 was used. Ex vivo infections utilized the mouse-adapted H1N1 influenza A virus strain HA-G222-mpJena/5258 [15]. To identify plaque-forming units (PFU), standard plaque assay was performed as described previously [16].

### Induction of cellular senescence

Cells were treated with recombinant human TNF-α (Thermo Fisher Scientific, Waltham, USA) and doxorubicin (TOCRIS, Bristol, UK) [17]. The medium of TNF-α-treated samples was changed every 2 d with freshly added TNF-α at a concentration of 20 ng/ml. Doxorubicin was added at a concentration of 250 nM to the IMR-90s. 24 h later a medium change with DMEM (10 % FBS, 1 % P/S) was performed, subsequently every 2 d. After 10 d of senescence induction, senescence-associated β-galactosidase staining (SA β-gal staining, Biovision, Cambridge, UK), according to the manufacturer’s protocol, was performed. Quiescent cells were used as negative controls and were derived from the same cell culture flask as the IMR-90s for senescence induction. Quiescence was prepared by serum arrest reducing the serum level to 0.2 % FBS for 2 d and then to 0 % FBS for 24 h [17].

### In vitro infections

After macrophage differentiation, cells were infected with the virus strain PR8 (influenza A virus/IAV/H1N1/Puerto Rico/8/1934) and a multiplicity of infection (MOI) 5. After washing the cells with phosphate-buffered saline (PBS, Thermo Fisher Scientific), they were incubated with the virus solution diluted in PBS (containing 0.2 % bovine serum albumin (BSA, Roth, Karlsruhe, Germany), 1 mM MgCl2 (Sigma Aldrich), and 0.9 mM CaCl2 (Sigma Aldrich)). After 30 min, the cells were washed with PBS and cultivated in Roswell Park Memorial Institute (RPMI, Thermo Fisher Scientific) medium (containing 0.2 % human serum albumin (HSA, PAN Biotech), 1 mM MgCl2, 0.9 mM CaCl2, and 30 ng/ml TPCK-treated trypsin (Thermo Fisher Scientific)) for 8 h and 24 h. Supernatants of infected hMDM were collected and used for standard plaque assay to determine the viral load and to determine cytotoxicity by using LDH assay (Thermo Fisher Scientific) according to the manufacturer’s protocol. PR8 was used to infect IMR-90s with an MOI 1; the cells were washed with PBS and incubated in virus solution diluted with PBS (containing 0.2 % BSA, 1 mM MgCl2, 0.9 mM CaCl2). After washing, the cells were cultivated in DMEM containing 0.2 % BSA, 1 mM MgCl2, 0.9 mM CaCl2, and 30 ng/ml TPCK-treated trypsin for 8 h and 24 h.

### Preparation of macrophage-conditioned medium

After the infection of hMDM with PR8 (MOI 5) for 24 h, supernatants were collected. The supernatants of infected hMDM were irradiated with UV light for 30 min to inactivate active virus particles [18]. The irradiated CM was mixed with fresh DMEM (10 % FBS, 1 % P/S) at a ratio of 1:1 and subsequently used for the cultivation of IMR-90s. Every 2nd day, the medium was diluted with CM and changed. After 5 d of stimulation, cellular senescence was confirmed by SA β-gal staining. Quiescent cells were prepared as described in the section “Induction of cellular senescence”.

### In vivo infections

5-week-old female BALB/cJRj-wild-type mice (Janvier, Le Genest-Saint-Isle, France, local ethics committee of the Thuringian State Office for Consumer Protection (TV-Nr: 02-018/16) were housed according to the institutional guidelines. Mice were anesthetized with 2 % isoflurane and infected intranasally with 10 µl virus solution per nostril and a viral titer of 106 PFU/ml (IAV/H1N1/ vi0084/2016). During the infection period, the body weight, general conditions, and spontaneous behavior of each animal were monitored daily for 21 d according to the scoring system (Supplementary Fig. 1A). The mice were sacrificed by an overdose of ketamine and xylazine.

### Ex vivo lung slices

Animal experiments were carried out under the approval of the local ethics committee of the Thuringian State Office for Consumer Protection (twz05-2022). C57/bl6 mice were euthanized with an overdose of ketamine and xylazine. After bleeding occurred from the axillary artery, 1.5 - 2 ml of a mixture of DMEM/F12 phenol-red free medium (Thermo Fisher Scientific) and 4 % agarose (Thermo Fisher Scientific) was injected intratracheally into the lungs of the mice under sterile conditions. The lungs were extracted and stored in PBS containing 1 % HEPES (Thermo Fisher Scientific). Left and right inferior lung lobes were cut with a vibratome (Leica VT1200S, Leica, Illinois, USA) into 300 µM thick sections and cultivated in DMEM/F12, GlutaMAX (Thermo Fisher Scientific) supplemented with 10 % FBS in 12-well plates. The cultivation medium was changed after 24 h, then every 2 d. Ex vivo lung slices were treated with recombinant murine TNF-α (ImmunoTools, Friesoythe, Germany) at a concentration of 20 ng/ml diluted in DMEM/F12 + 10 % FBS for 8 d. For infection, the slices were incubated with 5x105 PFU/ml of the H1N1 strain HA-G222-mpJena/5258 for 2 h, washed with PBS, and cultivated with DMEM/F12 (0.2 % BSA, 1 mM MgCl2, 0.9 mM CaCl2, and 30 ng/ml TPCK-treated trypsin) for 8 d. Medium was changed for both TNF-α-treated and IAV-infected slices every 2 d with DMEM/F12 + 10 % FBS.

### RNA Extraction, cDNA Synthesis, and qRT-PCR

Cultured cells were lysed, and lung tissues were homogenized in RLT buffer. Total RNA was isolated using the Qiagen RNeasy Mini Kit (Qiagen, Hilden, Germany) according to the manufacturer’s protocol. The RNA concentration and purity were determined using a NanoDrop spectrophotometer ND-1000 (Peqlab/VWR, Radnor, USA). cDNA synthesis was performed using the High-Capacity cDNA Reverse Transcription Kit (Thermo Fisher Scientific). Maxima SYBR Green qPCR Master Mix (Thermo Fisher Scientific) and Rotor-Gene Q (Qiagen) were used for qRT-PCR (95 °C for 10 min, followed by 45 cycles of 95 °C for 10 s, 60 °C for 20 s, and 72 °C for 30 s). β-Actin was used as the housekeeping gene, and the expression of targeted genes (metabion, Planegg, Germany) was normalized to it.

### Protein extraction and Western Blot

Cells were lysed in RIPA buffer (Thermo Fisher Scientific), vortexed, and centrifuged at 10.000 x g at 4 °C for 10 min. Protein concentrations were determined using Micro BCA Protein Assay Kit (Thermo Fisher Scientific). ). Extracted proteins were run on SDS-PAGE and blotted onto polyvinylidene fluoride (PVDF, Thermo Fisher Scientific) membranes. Following antibodies at a concentration of 1:1000 were used: β-Actin (Sigma Aldrich), p21 (Cell Signaling, Danvers, USA), and DPP4 (Cell Signaling). Subsequently, the membranes were incubated with the corresponding horseradish peroxidase (HRP)-conjugated anti-rabbit and anti-mouse IgG secondary antibody (bio-rad, Hercules, USA) at a concentration of 1:1000. Visualization was performed using Immobilon Western chemiluminescent HRP substrate (Merck Millipore, Darmstadt, Germany) on a FusionFX imaging device (Vilber, Collégien, France).

### Immunocytochemistry and Immunofluorescence

IMR-90s and hMDM were cultured on coverslips in 24-well plates, fixed with 4 % paraformaldehyde (PFA, Sigma Aldrich) for 30 min at 37 °C, permeabilized with 0.1 % Triton-X (Roth) in PBS, and blocked with 3 % BSA in PBS at 20-22 °C for 30 min. Coverslips were incubated with primary antibodies IAV nucleocapsid protein (abcam, Cambridge, UK, 1:200), CD68 (Thermo Fisher Scientific, 1:200), and Alexa Fluor Plus 647 phalloidin (Thermo Fisher Scientific, 1:400) diluted in blocking solution at 4 °C overnight. Alexa Fluor 488 AffiniPure Donkey Anti-Rabbit IgG, Cy3 AffiniPure Donkey Anti-Mouse IgG, Cy3 AffiniPure Donkey Anti-Rabbit IgG, and Alexa Fluor 488 AffiniPure Goat Anti-Mouse (Jackson Immuno Research, Cambridge, UK, 1:500) solutions were used as secondary antibodies. Mounting was performed using DAPI Fluoromount-G (Southern Biotech, Birmingham, USA) and visualization using an AxioObserver Z.1+Apotome 2 (Zeiss, Jena, Germany).

For histology of the in vivo experiment, samples were shock-frozen and cut to a thickness of 4 µm by using a Leica CM1950 microtome (Leica). The air-dried sections were placed on Superfrost Plus Adhesion Microscope Slides (Thermo Fisher Scientific) and stored at - 20 °C. For immunofluorescence, the slides were fixed in ice-cold acetone and incubated with the primary antibodies against p21 (Thermo Fisher Scientific, 1:200), p16 (Thermo Fisher Scientific, 1:200), and DPP4 (Thermo Fisher Scientific, 1:200) at 4°C overnight. Next, the secondary antibodies Alexa Fluor 488 Donkey Anti-Rabbit (Jackson Immuno Research, 1:500) and BODIPY 558/568 phalloidin (Thermo Fisher Scientific, 1:400) were applied to the slides. Tissue sections were mounted with DAPI Fluoromount-G (Southern Biotech) and observed with an AxioObserver Z.1+Apotome 2 (Zeiss).

The immunostaining images were validated with specific isotypes for rabbit (DA1E) mAb IgG XP. In addition, images of infected and uninfected, with and without antibodies were obtained as controls.

### Flow Cytometry

LEGENDplex (BioLegend, San Diego, USA) assay was performed according to the manufacturer’s protocol. The kits LEGENDplex Human Inflammation Panel I, Human Macrophage/Microglia Panel, and Mouse Anti-Virus Response Panel were used. All specimens were analyzed using an Accuri C6 Plus Cytometer (BD Biosciences, Heidelberg, Germany) and calculated using the data analysis software LEGENDplex v8.0.

### mRNA Sequencing

mRNA sequencing was performed at Novogene (Novogene, Peking, China). Briefly, RNA was extracted, and quantification was performed using Qubit 4 fluorometer (Thermo Fisher Scientific). For mRNA sequencing, a 250-300 bp insert cDNA library was prepared using the NEB Next Ultra RNA Library Prep Kit. RNA sequencing was performed on the Illumina platform Novaseq 6000 S4 flowcell V1.0, based on the mechanism of sequencing by synthesis (SBS) and the PE150 strategy. For bioinformatics, raw reads in FASTQ format were first processed using fastp; clean data were generated by removing reads containing adapters and poly N sequences, as well as reads with low quality from the raw data. Reference genome and gene model annotation files were downloaded directly from the genome website browser (NCBI/UCSC/Ensembl). The clean paired-end reads were mapped to the reference genome using the HISAT2 software. Differential gene expression analysis was determined by using the DESeq2 software and negative binomial distribution. The Benjamini-Hochberg procedure was used as FDR calculation method.

Preparation of the heatmaps was generated using R (v3.6.3, R Foundation for Statistical Computing).

### Statistical analysis and scheme design

Statistical analyses were performed using the GraphPad Prism 9 software (GraphPad, San Diego, USA) with Mann-Whitney U test, One-way ANOVA, and Two-way ANOVA (ns p > 0.05; * p ≤ 0.05; ** p ≤ 0.01; *** p ≤ 0.001; **** p ≤ 0.0001). The figures were designed using Biorender.com.

## RESULTS

### IAV infection induces cellular senescence in murine lungs

**Figure 1.**
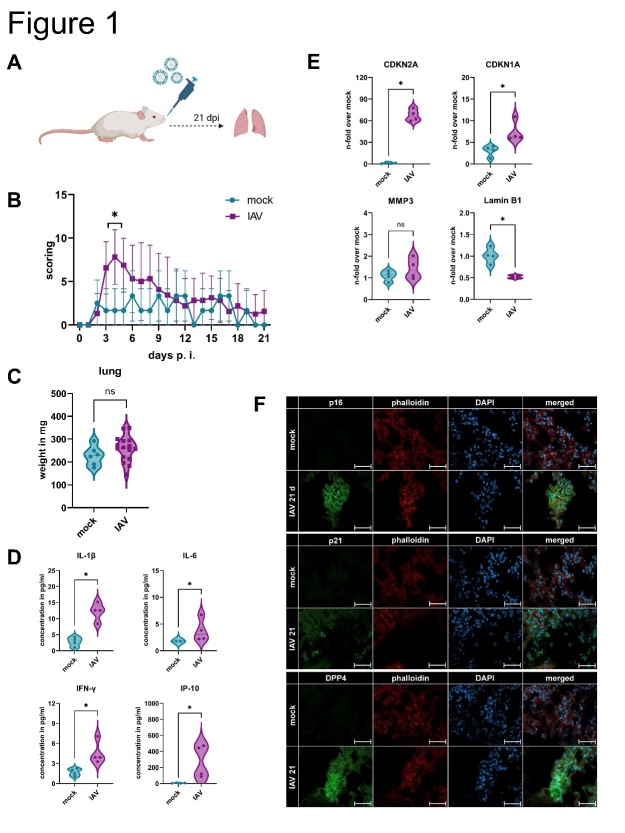
**IAV infection induces cellular senescence in murine lungs**. (**A**) Schematic representation of IAV infection of BALB/c mice for 21 d. (**B**) Overall evaluation of IAV-infected mice for 21 d revealed the highest scoring on day 4 (N ≥ 6). Significance was calculated with Two-way ANOVA (* p ≤ 0.05). (**C**) After euthanasia, the weight of the lung was determined in mg (N ≥ 6). Significance was calculated with the Mann-Whitney U test (ns p > 0.05). (**D**) Cytokine detection of homogenized lungs revealed upregulation of the pro-inflammatory factors IL-1β, IL-6, IFN-γ, and IP-10 adjusted to lung weight (N = 4). Significance was calculated with the Mann-Whitney U test (* p ≤ 0.05). (**E**) qRT-PCR analysis of IAV-infected mice showed upregulation of CDKN2A and CDKN1A and downregulation of Lamin B1 (N = 4). Significance was calculated with the Mann-Whitney U test (ns p > 0.05; * p ≤ 0.05). (**F**) Immunofluorescence staining of histological lung sections for p16, p21, DPP4, phalloidin (actin filaments), and DAPI (nucleus). Scale bar 50 µm.

To investigate the impact of IAV infection on cellular senescence in the lungs, we used a murine infection model (Fig. 1A). Daily scoring of the body weight, general conditions, and spontaneous behavior revealed that IAV-infected mice had a higher score compared to the mock-infected mice on day 4 (Fig. 1B, Supplementary Fig. 1A). After 21 d the condition of the lungs of the infected mice was similar to that of the mock-infected mice, i.e., with total clearance of the virus, suggesting recovery from IAV infection (Fig. 1C, Supplementary Fig. 1B). Interestingly, 21 d after IAV infection, the secretion of the pro-inflammatory cytokines IL-1β, IL-6, interferon (IFN)-γ, and IFN-gamma-induced protein (IP)-10 was increased (Fig. 1D). qRT-PCR analysis revealed significant upregulation of cellular senescence-related genes in the lungs, namely cyclin-dependent kinase inhibitor 2A (CDKN2A), and CDKN1A, whereas Lamin B1 was significantly downregulated, which is a characteristic of senescent cells (Fig. 1E). To validate the presence of senescence-associated proteins in the lungs of infected mice, we performed immunofluorescence staining and obtained positive signals for p16, p21, and dipeptidyl peptidase 4 (DPP4) in IAV-infected samples compared to those in mock-infected samples (Fig. 1F).

**Figure 2.**
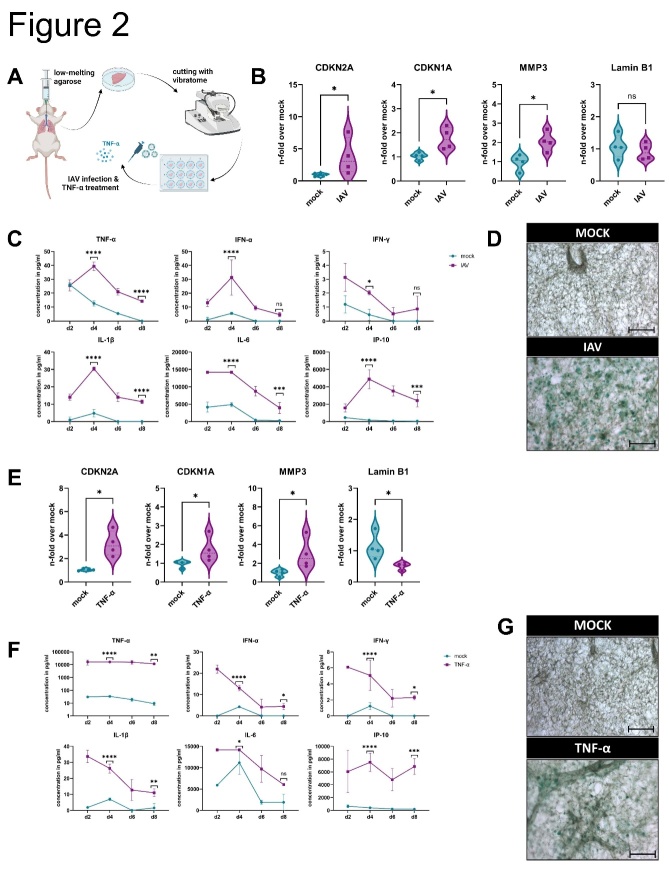
**Influenza virus infection of ex vivo lung slices results in premature cellular senescence**. (**B-D**) IAV infection, (**E-G**) TNF-α stimulation. (**A**) Schematic representation of the preparation of *ex vivo* lung slices. (**B, E**) *Ex vivo* lung slices were infected with IAV or treated with recombinant murine TNF-α for 8 d. qRT-PCR analysis was performed for CDKN2A, CDKN1A, MMP3, and Lamin B1 (N = 4). Significance was calculated with the Mann-Whitney U test (ns p > 0.05; * p ≤ 0.05). (**C, F**) After IAV infection and TNF-α treatment, the pro-inflammatory factors TNF-α, IFN-α, IFN-γ, IL-1β, IL-6, and IP-10 were upregulated on day 4 (N= 3). Significance was calculated with Two-way ANOVA (ns p > 0.05; * p ≤ 0.05; ** p ≤ 0.01; *** p ≤ 0.001; **** p ≤ 0.0001). (**D, G**) *Ex vivo* lung slices were stained with SA β-gal staining after 8 d of mock and IAV infection as well as 8 d of TNF-α treatment. Scale bar 200 µm.

### Influenza virus infection of ex vivo lung slices results in premature cellular senescence

To confirm our findings, we performed a targeted examination of murine ex vivo lung slices infected with IAV (Fig. 2A-D). To determine the expression of cellular senescence-related mRNA, we analyzed CDKN2A, CDKN1A, MMP3, and Lamin B1 in the slices 8 d post infection (Fig. 2B). We observed a significant upregulation of CDKN2A, CDKN1A and matrix metallopeptidase 3 (MMP3) in the IAV-infected lung slices. Pro-inflammatory factors were upregulated on day 4 due to IAV infection, with the highest viral titer also observed on day 4 (Fig. 2C, Supplementary Fig. 2). Ex vivo lung slices were stained with SA β-gal after 10 d of IAV and mock infection, and the characteristic blue staining of senescent cells was visible in the IAV-infected slices (Fig. 1D).

Our findings indicate a strong inflammatory response after IAV infection with high TNF-α concentrations. To elucidate the impact of TNF-α, we stimulated murine ex vivo lung slices with recombinant TNF-α (Fig. 2E-G). After TNF-α stimulation for 8 d, a significant upregulation of CDKN2A, CDKN1A, and MMP3 as well as downregulation of Lamin B1 were detected (Fig. 2E). TNF-α stimulation resulted in the upregulation of pro-inflammatory cytokines (Fig. 2F). After 8 d of stimulation, SA β-gal staining revealed senescent cells in the lung slices (Fig. 2G).

### TNF-α production by macrophages during infection leads to cellular senescence in human lung fibroblasts

**Figure 3.**
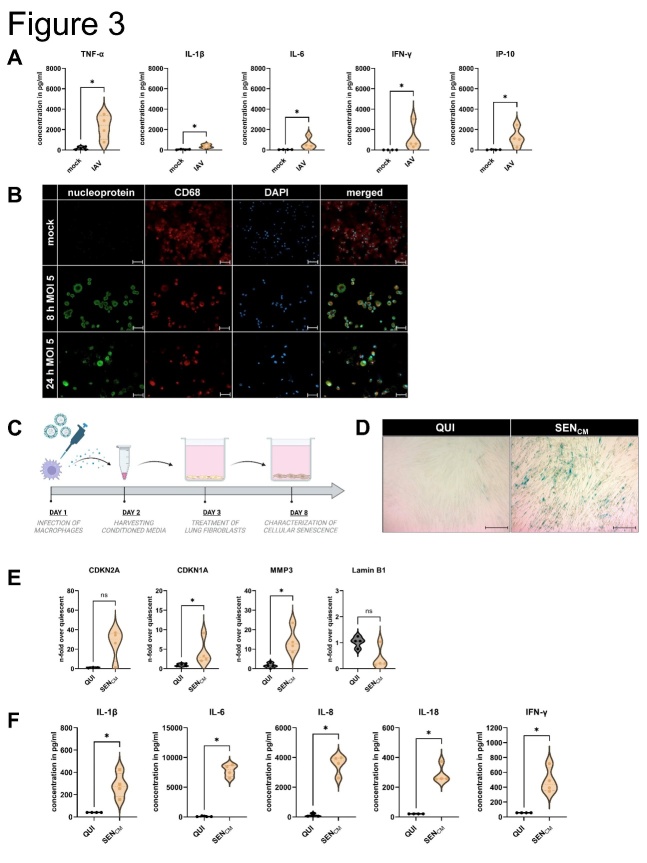
**TNF-α production by macrophages during infection leads to cellular senescence in human lung fibroblasts**. (**A**) Infection of the hMDM was performed with IAV (H1N1, PR8) for 8 h and 24 h and MOI 5. After 24 h of infection, hMDM produced high levels of the cytokines and chemokines TNF-α, IFN-γ, IP-10, IL-1β, and IL-6 (N = 4). Significance was calculated with the Mann-Whitney U test (* p ≤ 0.05). (**B**) Immunofluorescence staining of mock- and IAV-infected hMDM of IAV-specific nucleoprotein, CD68, and DAPI. Scale bar 50 µm. (**C**) Schematic representation of treatment of the IMR-90s with supernatants of IAV-infected hMDM (conditioned medium, CM). (**D**) Using SA β-gal staining, we detected a positive signal in the IMR-90s treated with conditioned media (SEN_CM_). Scale bar 200 µm. (**E**) After CM treatment (day 8), qRT-PCR analysis was performed, and CDKN1A, and MMP3 were significantly increased. Significance was calculated with the Mann-Whitney U test (ns p > 0.05; * p ≤ 0.05). (**F**) Protein levels were determined, and the pro-inflammatory factors IL-1β, IL-6, IL-8, IL-18, and IFN-γ were significantly upregulated (N = 4). Significance was calculated with the Mann-Whitney U test (* p ≤ 0.05).

To investigate the mechanism underlying virus-induced cellular senescence, we infected hMDM with PR8, MOI 5 (PFU 6.5 PFU/ml) for 8 h and 24 h. Here, high levels of cytokines and chemokines (TNF-α, IFN-γ, IP-10, IL-1β, and IL-6), with significantly upregulated TNF-α levels 24 h after infection could be detected (Fig. 3A). The infection was confirmed by immunofluorescence staining of IAV-related proteins and macrophage specific markers and the standard plaque assay (Fig. 3B, Supplementary Fig. 3A). Moreover, the cytotoxicity induced by IAV was significantly increased 24 h after infection (Supplementary Fig. 3B). Cytotoxicity was determined from the supernatant of macrophages by using LDH assay (Thermo Fisher Scientific).

**Figure 4.**
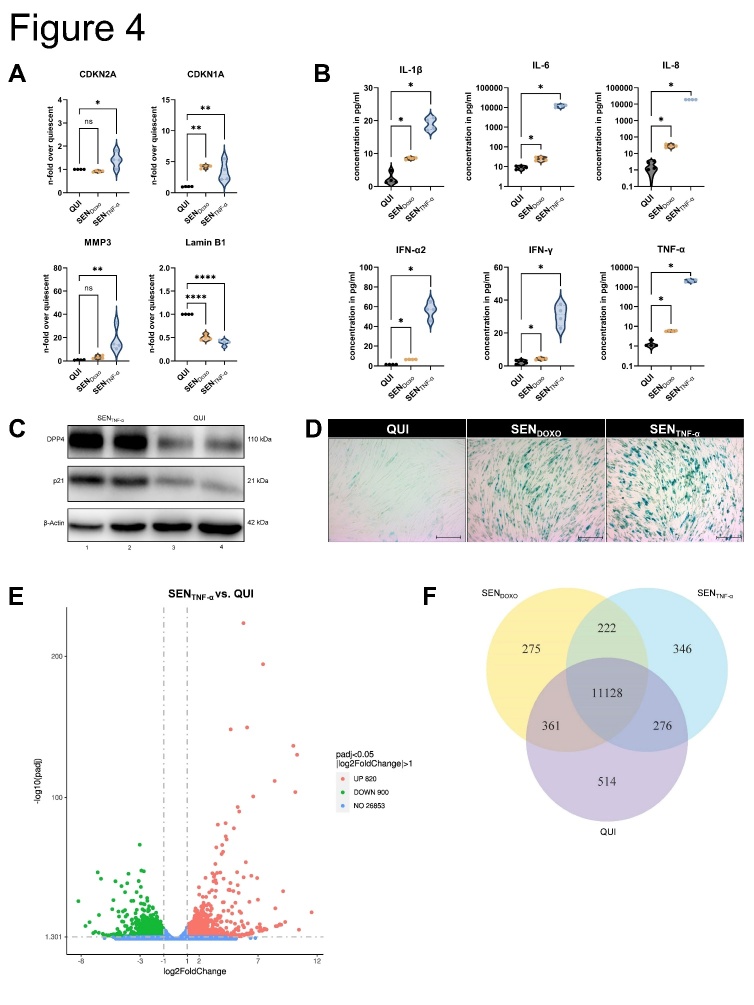

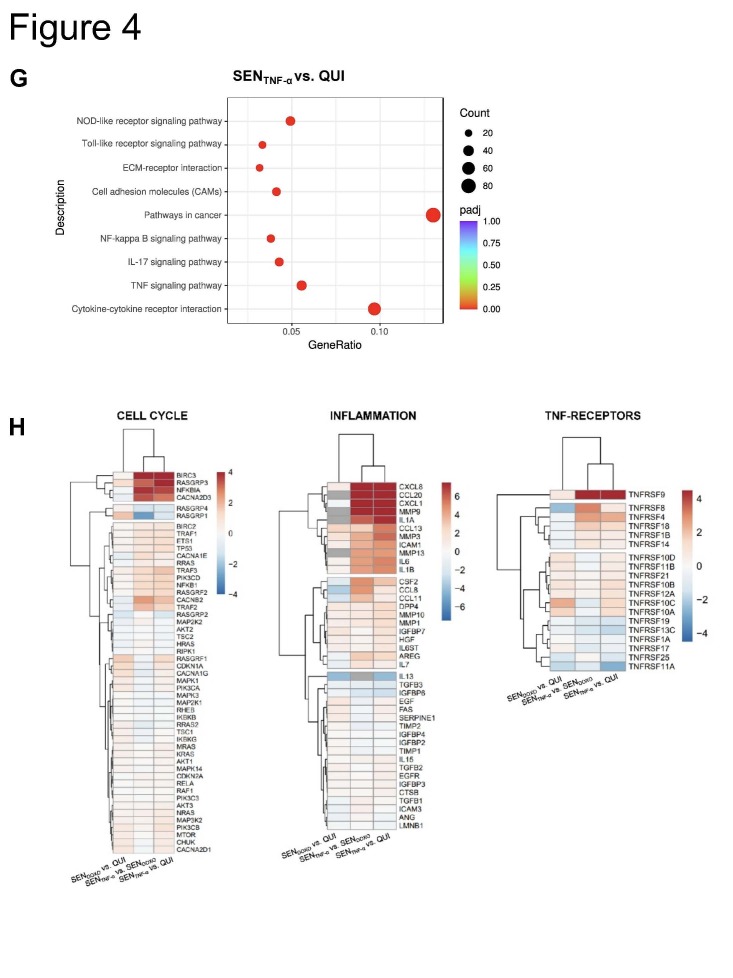

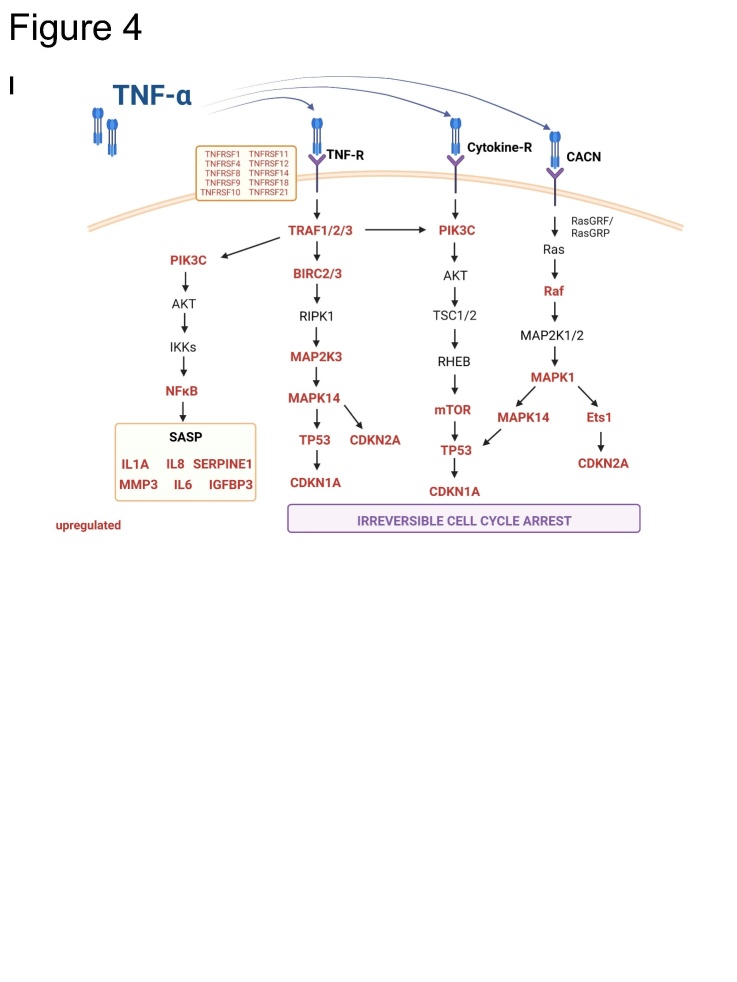
**Recombinant human TNF-α induces cellular senescence in human lung fibroblasts**. (**A**) The induction of cellular senescence was performed by using doxorubicin (SEN_DOXO_) or TNF-α (SEN_TNF-α_) compared to quiescent cells (QUI) for 10 d. The mRNA expression of CDKN2A, CDKN1A, and MMP3 was increased, whereas Lamin B1 was decreased in SEN_TNF-α_ (N = 4). Significance was calculated with One-way ANOVA (ns p > 0.05; * p ≤ 0.05; ** p ≤ 0.01; **** p ≤ 0.0001). (**B**) IL-1β, IL-6, IL-8, IFN-α2, IFN-γ, and TNF-α exhibited an increase during TNF-α treatment of IMR-90s (N = 4). Significance was calculated with the Mann-Whitney U test (* p ≤ 0.05). (**C**) The expression of the senescence-associated proteins DPP4 and p21 was determined by using western blot analysis in SEN_TNF-α_ (N = 3). (**D**) The SA β-gal staining showed blue coloring in SEN_DOXO_ and SEN_TNF-α_, which is related to senescence_._ Scale bar 200 µm. (**E**) mRNA transcriptomic analysis of IMR-90s was performed after senescence induction with TNF-α (SEN_TNF-α_) and doxorubicin (SEN_DOXO_) compared to quiescent cells (QUI). Significantly differential regulated RNAs are shown as a volcano plot and (F) Venn diagram. (**G**) KEGG enrichment analysis showed upregulated signaling pathways related to the cell cycle and cytokine-cytokine receptor interaction of SEN_TNF-α_ compared to QUI. (**H**) Individually regulated genes are illustrated in heatmaps regarding cell cycle (left panel), inflammation (middle panel), and TNF receptors (right panel). Gene expression in heatmaps over log2 fold-change. (**I**) Schematic representation of the influence of TNF-α on human lung fibroblasts via various pathways.

We determined the relationship between TNF-α production by hMDM and the induction of cellular senescence in IMR-90s using macrophage-conditioned medium (CM) irradiated with UV-light to inactivate virus particles (Fig. 3C). Compared with quiescent cells (QUI), a higher number of SA β-gal positive cells were observed among the senescent cells induced by CM treatment (SENCM) (Fig. 3D). qRT-PCR analysis of CM-treated IMR-90s revealed a significant upregulation of CDKN1A and MMP3, (Fig. 3E). We analyzed the supernatants of the CM-treated IMR-90s and observed significant upregulation of IL-1β, IL-6, IL-8, IL-18, and IFN-γ (Fig. 3F). We confirmed our findings by the stimulation of IMR-90s with TNF-α (SENTNF-α) (Fig. 4A-F). QUI were used as negative control, and senescence induction using Doxorubicin (SENDOXO) as a positive control.

qRT-PCR analysis revealed the upregulated mRNA expression of cellular senescence-related parameters; the expression of CDKN2A, CDKN1A, and MMP3 was significantly upregulated, whereas Lamin B1 was significantly downregulated in SENTNF-α (Fig. 4A). Moreover, SASP-related proteins were significantly upregulated (Fig. 4B). These findings were verified at the protein level using western blotting for DPP4 and p21 and via the results of SA β-gal staining (Fig. 4C, D).

Afterwards, we performed mRNA sequencing of QUI cells, SENDOXO, and SENTNF-α (Fig. 4E). A total of 222 genes were common between the SENDOXO and SENTNF-α cells, whereas 346 were individually regulated in SENTNF-α (Fig. 4F, Supplementary Table 1). Specific pathways associated with cancer, cytokine-cytokine receptor interactions, and chemotaxis were highly upregulated in the SENTNF-α (Fig. 4G, Supplementary Fig. 4A). Individually regulated genes are shown in the heat map (Figure 4H). Based on KEGG pathway analysis, a similar trend of gene expression was observed between SENDOXO vs. QUI, and between SENTNF-α vs. QUI for cell cycle related genes (Fig. 4H, left panel). Here, e.g., the expression of Ras protein-specific guanine nucleotide-releasing factor 1 (RASGRF1), CDKN1A, mitogen-activated protein kinase 1 (MAPK1), and phosphatidylinositol-4,5-bisphosphate 3-kinase catalytic subunit alpha (PIK3CA) could be illustrated. However, a differential expression between SENDOXO and SENTNF-α, could be observed for genes encoding baculoviral IAP repeat containing 3 (BIRC3), Ras guanyl-releasing protein 3 (RASGRP3), NFKB inhibitor alpha (NFKBIA), calcium voltage-gated channel auxiliary subunit alpha2 delta 3 (CACNA2D3), and further cell cycle regulating genes.

Inflammatory genes exhibited similar expression patterns in both SENDOXO and SENTNF-α groups, e.g., upregulation of genes encoding insulin-like growth factor binding protein 7 (IGFBP7), epidermal growth factor (EGF), and Fas Cell Surface Death Receptor (FAS), whereas only SENTNF-α showed strong upregulation of genes encoding C-X-C motif chemokine ligand 8 (CXCL8), CXCL1, C-C motif chemokine ligand 20 (CCL20), MMP3, intercellular adhesion molecule 1 (ICAM1), and IL-6 (Figure 4H, middle panel) (Fig. 4H, middle panel).

SENDOXO and SENTNF-α treatment led to the upregulation of different TNF-α receptors (Fig. 4H, right panel). SENDOXO treatment led to the upregulation of TNF receptor superfamily member 10C (TNFRSF10C). In the SENTNF-α group, the following receptors were upregulated compared to their expression in the SENDOXO group: TNFRSF9, TNFRSF8, TNFRSF4, TNFRSF18, TNFRSF1B, and TNFRSF14. Thus, TNF-α stimulation influences the human lung fibroblasts via various receptors (Fig. 4I).

### Influenza virus load is enhanced in senescent human lung fibroblasts

**Figure 5.**
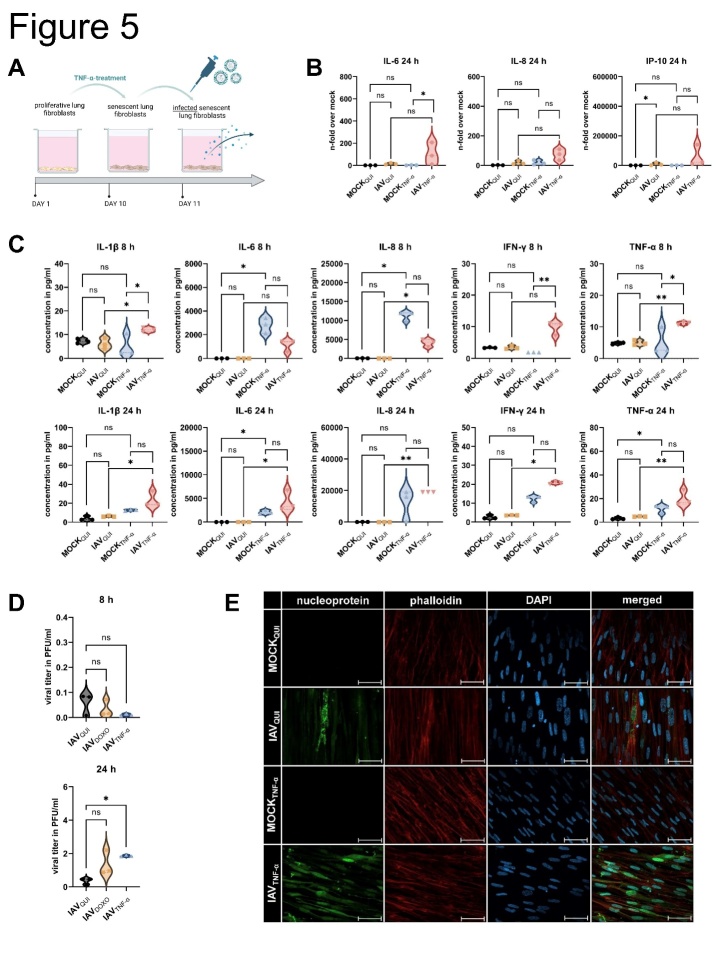

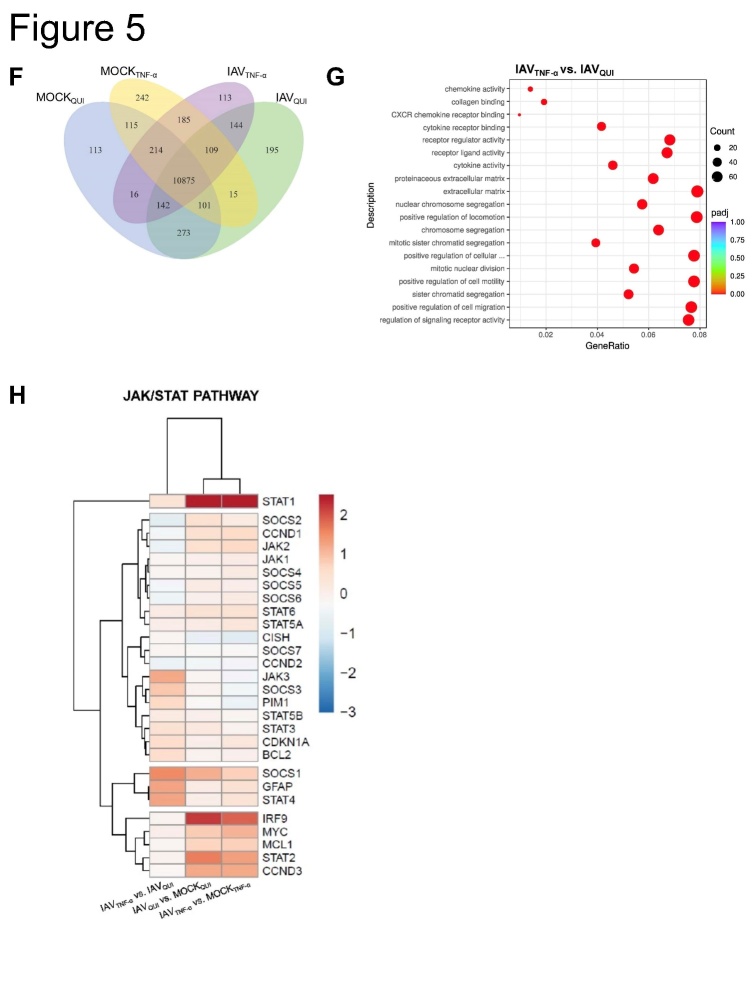

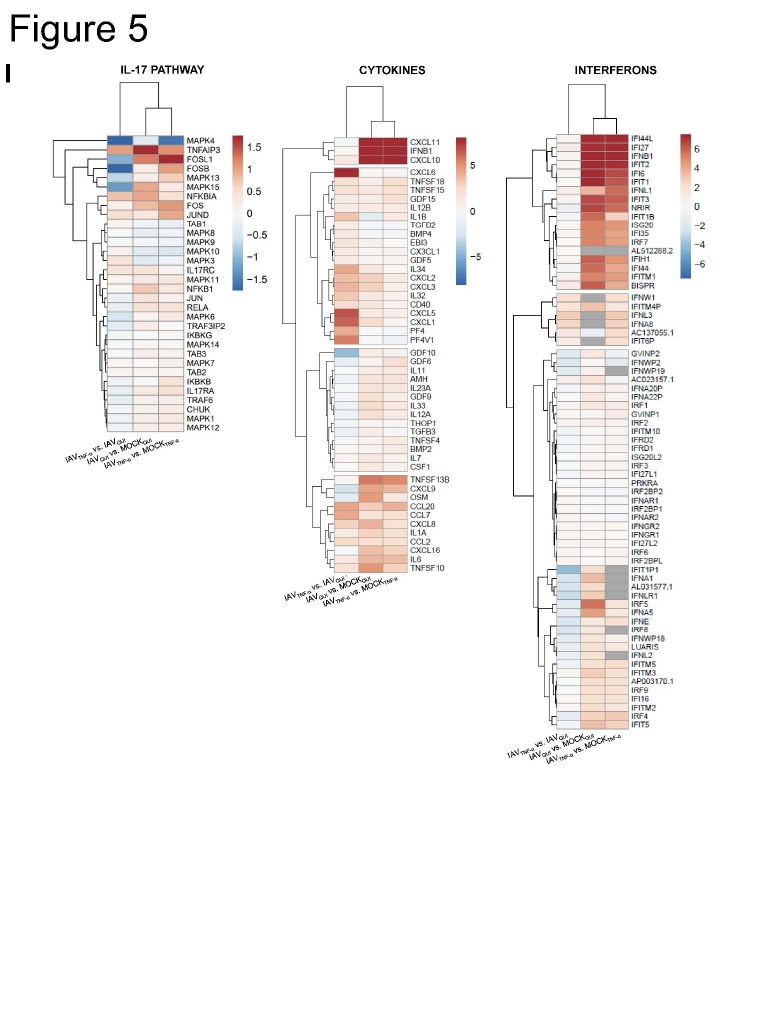

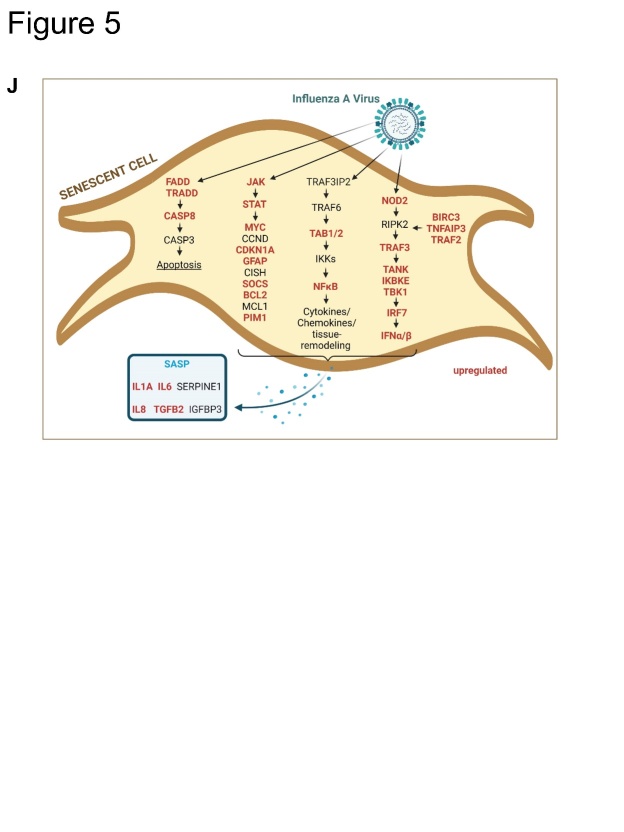
**Influenza virus load is enhanced in senescent human lung fibroblasts**. (**A**) Schematic representation of infection with IAV (MOI 1) of senescent IMR-90s (IAV_TNF-α_) for 8 h and 24 h. (**B**) qRT-PCR analysis of IL-6, IL-8, and IP-10 of quiescent mock- (MOCK_QUI_) and IAV-infected (IAV_QUI_), as well as uninfected senescent cells (MOCK_TNF-α_) and IAV_TNF-α_, was performed (N = 3). Significance was calculated with One-way ANOVA (ns p > 0.05; * p ≤ 0.05). (**C**) Protein levels of IL-1β, IL-6, IL-8, IFN-γ, and TNF-α were increased in IAV_TNF-α_ after 24 h (N = 3). Significance was calculated with One-way ANOVA (ns p > 0.05; * p ≤ 0.05). (**D**) Viral titer detected by standard plaque assay was increased in IAV_TNF-α_ compared with IAV_QUI_ after 24 h (N = 3). Significance was calculated with One-way ANOVA (ns p > 0.05; * p ≤ 0.05). (**E**) Immunofluorescence staining of IAV_TNF-α_ and IAV_QUI_ for IAV nucleoprotein, phalloidin, and DAPI showed higher signals for IAV_TNF-α_. Scale bar 50 µm. (**F**) Transcriptomic analysis of senescent (IAV_TNF-α_) and quiescent IMR-90s (IAV_QUI_) after IAV infection. Overall, in the Venn diagram, the IAV_TNF-α_ group shares 144 RNAs with the IAV_QUI_ group. 113 RNAs are individually expressed in IAV_TNF-α_ in comparison to 195 individually expressed RNAs in the IAV_QUI_. (**G**) Different signalling pathways are illustrated in the GO enrichment analysis of IAV_TNF-α_ and IAV_QUI_. The IAV_TNF-α_ group has significant changes regarding cell motility, extracellular matrix, and signalling receptor activity. (**H, I**) Individually expressed genes are demonstrated in heatmaps. The genes JAK3, SOCS3, SOCS1, TNFAIP3, and CXCL6, are differentially expressed in IAV_TNF-α_ compared to IAV_QUI_. Gene expression in heatmaps over log2 fold-change. (**J**) Schematic representation of the influence of an IAV infection on senescent cells and the SASP.

To identify the consequences of the induction of cellular senescence in the lung, we infected TNF-α-treated IMR-90 cells with IAV for 8 h and 24 h and MOI 1 (Fig. 5A). The treatment with TNF-α without infection (MOCKTNF-α) did not result in the upregulation of IL-6, IL-8, and IP-10 on mRNA level at either 8 h or 24 h (Fig. 5B, Supplementary Fig. 5A). IAV infection of senescent cells (IAVTNF-α) compared to quiescent cells (IAVQUI) led to the significant induction of IL-8 after 8 h. At 24 h after IAV infection, the comparison between IAVTNF-α and IAVQUI revealed the tendency of increased cytokines IL6, IL-8, and IP-10.

After IAV infection, the protein levels of the supernatants of MOCKQUI and IAVQUI, as well as MOCKTNF-α and IAVTNF-α were determined using Legendplex analysis (Fig. 5C). TNF-α treatment of mock samples showed an increased protein level of IL-6 and IL-8 after 8 h, and IL-6 and TNF-α after 24 h compared to MOCKQUI. However, subsequent virus infection of TNF-α treated cells demonstrated a significant increase of IL-1 β, IFN-γ, and TNF-α after 8 h measured on the protein levels. IAVTNF-α demonstrated significantly higher levels of IL-6, IL-1β, IL-8, IFN-γ, and TNF-α in comparison to IAVQUI after 24 h infection.

The standard plaque assay revealed that the viral load was significantly increased in IAVTNF-α after 24 h of infection (Fig. 5D). Consistent with this finding, the immunofluorescence staining of IAVQUI and IAVTNF-α confirmed the upregulation of IAV-specific nucleoprotein in IAVTNF-α (Fig. 5E).

To uncover the pathomechanisms underlying the occurrence of higher viral titers in IAVTNF-α, we conducted mRNA sequencing of IAVQUI, IAVTNF-α, and quiescent and senescent IMR-90s without IAV infection (MOCKQUI and MOCKTNF- α). The Venn diagram presents all differentially expressed genes, revealing the differences in intracellular regulation between IAVTNF-α and IAVQUI (Fig. 5F). The results revealed that 144 genes were commonly regulated in both conditions, whereas only 113 and 195 genes were differentially regulated in IAVTNF-α cells and IAVQUI cells, respectively. Here, the upregulation of specific pathways in IAVTNF-α and IAVQUI was identified (Fig. 5G). The highest differential expression was observed for the following pathways: extracellular matrix, positive regulation of locomotion, cellular component movement, and cell motility as well as regulation of signaling receptor activity.

We observed differential regulation of the following genes between IAVTNF-α and IAVQUI cells: JAK3, suppressor of cytokine signaling 3 (SOCS3), SOCS1, glial fibrillary acidic protein (GFAP), and STAT4 (Fig. 5H).

In the IAVTNF-α group compared to IAVQUI, the transcription factors Jun, FOS like 1 (FOSL1), FOSB, and mitogen-activated protein kinase (MAPK) 4, 13, and 15 were downregulated, whereas TNF alpha-induced protein 3 (TNFAIP3, A20) was upregulated (Fig. 5I, left panel). Several genes, including CXCL11, IFNB1, CXCL10, CXCL9, and IL-6 were equally upregulated by IAV infection in senescent and quiescent cells compared to their mocks (Fig. 5I, middle panel). TNFSF10 (TRAIL) revealed an upregulation in IAVTNF-α infected cells compared to IAVQUI (Supplementary Figure 5B, left panel). The chemokines CXCL1, CXCL5, and CXCL6 were significantly upregulated between IAVTNF-α and IAVQUI. In contrast, IFN signaling is mainly dependent on IAV infection and is independent of TNF-α-induced senescence (Fig. 5I, right panel). Among the IFNs, only IFNA8 and IFNB1 were upregulated in senescent IAV-infected cells (Supplementary Fig. 5B, right panel).

To identify the processes activated in infected, senescent cells, we analyzed different intracellular pathways using mRNA-seq. We observed the upregulation of the JAK/STAT, NOD, and NF-κB signaling pathways, resulting in the upregulation of cytokines and chemokines (Fig. 5J).

## DISCUSSION

In this study, we analyzed the effect of IAV infection on the induction of cellular senescence. Respiratory viral infections lead to both a direct cytopathic effect and a paracrine-induced, inflammation-triggered impact on the environment. To analyze the relationship between aging and virus infection, we used both murine and human specific models of IAV infection exhibiting characteristics of cellular senescence. Different mouse models and virus strains were used to gain a more sophisticated insight and to explore the changes within the host in a strain-independent manner. To the best of our knowledge, we are the first to indicate that there is a paracrine link between viral infection and the induction of premature cellular senescence.

Our murine model of IAV infection exhibited an expected course of disease with a scoring maximum on day 4 (Figure 1). The obtained data clearly indicates a clearance of infectious virus after 21 days. However, mRNA expression and immunohistochemistry of senescence-associated markers indicate an effect on the cell cycle. In addition, SASP-related inflammatory parameters were upregulated.

In order to distinguish between infection-associated effects on the cell cycle and the establishment of cellular senescence, further studies, in particular on the long-term course of the infection, would be imperative.

Lung fibroblasts can be infected with IAV and are considered to play a crucial role in the establishment of severe lung injury [19]. The initial infection of pneumocytes in the alveoli leads to activation of alveolar macrophages, thereby affecting lung fibroblasts as well; however the homeostasis between fibroblasts and macrophages is mainly studied for lung fibrosis [20].

Since alveolar macrophages are an important first defense mechanism in respiratory viral infections, we investigated their importance as a link between virus and lung fibroblasts. Macrophages are known as the major producers of TNF-α [21], and our results revealed a massive upregulation of TNF-α after IAV infection. We used the CM of infected macrophages to stimulate IMR-90 cells and observed a significant increase in the levels of cellular senescence-associated parameters including SASP factors (Fig. 3).

To analyze the mechanism underlying CM-induced cellular senescence, we stimulated primary human lung fibroblasts with TNF-α (Fig. 4). At the protein level and as per SA β-gal staining, TNF-α-induced cellular senescence was confirmed (Fig. 4C, D). Moreover, the expression of SASP-related factors confirmed a senescent phenotype. Differentially regulated genes indicate the upregulation of specific pathways associated with the cell cycle and cytokine-cytokine receptor interaction in SENTNF-α cells (Fig. 4G).

Previous studies have reported that the activation of TNF-α/IFN-γ results in hyperinflammation associated with senescence [22]. Our results clearly indicate an induction of cellular senescence via this pathway by the upregulation of various TNF-receptors (Fig. 4I).

To validate the involvement of the paracrine mechanism in IAV-triggered TNF-α- induced senescence, we used a murine ex vivo lung model (Fig. 2). This model enabled daily monitoring of the inflammatory course of the IAV-infected slices under the influence of the alveolar macrophages present in the lung tissue and premature cellular senescence could be verified.

However, to genuinely confirm the role of alveolar macrophages in contrast to interstitial macrophages in virus-induced cellular senescence, further studies focusing on alveolar macrophages should be performed.

After identifying the relationship between IAV infection and the induction of cellular senescence via premature paracrine TNF-α stimulation, we investigated the impact of subsequent IAV infections on senescent cells (Fig. 5A).

Simple stimulation of IMR-90s with TNF-α without infection (MOCKTNF-α) did not result in upregulation of the factors IL-6, IL-8, and IP-10 at mRNA level (Figure 5B, Supplementary Fig. 5A). However, IAV infection of IMR-90s (IAVTNF-α) revealed increased protein levels of pro-inflammatory cytokines and chemokines compared to those in IAVQUI (Figure 5C). This result can be explained by the upregulation of the transcription factors NF-κB, IRF, and STAT (Fig. 5H, Supplementary Fig. 5B) [23].

Moreover, the viral titer after 24 h of IAV infection was significantly increased compared to that in IAVQUI (Fig. 5D). Given the antiviral effects of cytokines secreted by IAVTNF-α cells, this result is unexpected. However, high antiviral cytokines and chemokines combined with high viral titers were also detected for other influenza A virus types (H5N1, H7N9) previously [24, 25].

To explain this increase, we considered (I) the impact of the pH on viral replication. pH is a critical parameter for viral replication because the uncoating of the viral nucleic acid is pH-dependent [26]. By using SA β-gal staining, we detected senescent cells with pH 6. This confirms a change of the pH-value [27]. It has been published previously that lower pH values increase viral replication [28].

Further, (II) changes in the signaling cascade are known to influence viral replication. Therefore, we performed mRNA-sequencing of IAVQUI and IAVTNF-α (Fig. 5F-J). The differentially expressed genes were mainly related to the JAK/STAT signaling pathway. Particularly, in the IAVTNF-α and IAVQUI cells, the genes JAK3, SOCS1, SOCS3, and STAT4 were differentially expressed. Moreover, in the IAVTNF-α group, senescent-infected samples exhibited upregulated TNFSF10/ TRAIL (Supplementary Fig. 5B, left panel). The role of TRAIL has been investigated in the context of influenza infection [29]. Previous studies reported that TRAIL deficiency leads to severe influenza infection and high viral titers [30]. Therefore, TRAIL acts as a proviral factor that enhances viral propagation [31]. It could be assumed that increased TRAIL expression results in an enhanced viral load, which is in accordance with the results of our study.

A previous study reported that NS1 increased the protein levels of TNFAIP3 (A20) [32], which is in agreement with the mRNA sequencing results of the current study, i.e., upregulation of A20 in the IAVTNF-α and IAVQUI cohorts (Fig. 5I, left panel).

Our study provides new insights into the mechanisms underlying aging and lung infection. We demonstrated that viral infections lead to premature cellular senescence via paracrine induction of TNF-α. The results contribute significantly to our understanding of the relationship between lung infections and aging, as subsequent viral infections lead to higher viral titers in senescent cells.

## Supplementary Materials


