# Supplementary material for: Influenza Virus-Induced Paracrine Cellular Senescence of the Lung Contributes to Enhanced Viral Load
Source: Aging Dis. 2023 Aug 1;14(4):1331–48. doi: 10.14336/AD.2023.0310 (PMC10389813; doi:10.14336/AD.2023.0310)
Supplement: Supplementary file 1 [file AD-14-4-1331-s.pdf]

SUPPLEMENTARY DATA

Influenza Virus-Induced Paracrine Cellular Senescence of the Lung Contributes to Enhanced Viral Load

Luise Schulz, Franziska Hornung, Antje Häder, Lukáš Radosa, Axel A Brakhage, Bettina Löffler[1], Stefanie Deinhardt-Emmer

© 2023. Schulz L et al. Published online at https://www.aginganddisease.org/EN/10.14336/AD.2023.0310

SUPPLEMENTARY DATA

A

CATEGORY	OBSERVATION	GRADING
**BODYWEIGHT**	Not affected	0
	Reduction of 5 – 10 %	5
	Reduction of > 10 %	10
	Reduction of > 15 %	15
	Reduction of > 20 %, for max. 48 h	20
**GENERAL CONDITION**	Shiny and smooth fur, clean orifices, normal eating and drinking behavior, normal urination and defecation	0
	Bluff and scrubby fur, dingy eyes, malfunctioning eating and drinking behavior, problems with urination and defecation	5
	Clotted orifices, partially closed eyes, dehydration, abnormal posture	10
	Closed eyes	20
**SPONTANEOUS BEHAVIOR**	No conspicuous features (calm and attentive, normal social behavior)	0
	Slightly different (very calm, limited motor function)	5
	Strongly altered (isolation, expression of pain, stereotypies, problems with coordination)	10
	Automutilation	20

B

ID	CONDITION	PFU/ML
**991**	IAV 21 dpi	0
**992**	IAV 21 dpi	0
**993**	IAV 21 dpi	0
**994**	IAV 21 dpi	0
**995**	IAV 21 dpi	0
**996**	IAV 21 dpi	0
**997**	IAV 21 dpi	0
**998**	IAV 21 dpi	0
**999**	IAV 21 dpi	0
**1006**	IAV 21 dpi	0
**1007**	IAV 21 dpi	0
**1008**	IAV 21 dpi	0
**1014**	IAV 21 dpi	0
**1015**	IAV 21 dpi	0
**1016**	IAV 21 dpi	0
**1017**	IAV 21 dpi	0
**1018**	IAV 21 dpi	0
**1019**	IAV 21 dpi	0

Supplementary Figure 1. (A) IAV infection was performed in BALB/c mice for 21 d. Scoring system to evaluate the health of mice. (B) Virus particles determined using standard plaque assay of lung homogenates were not detectable on day 21 (N = 18).

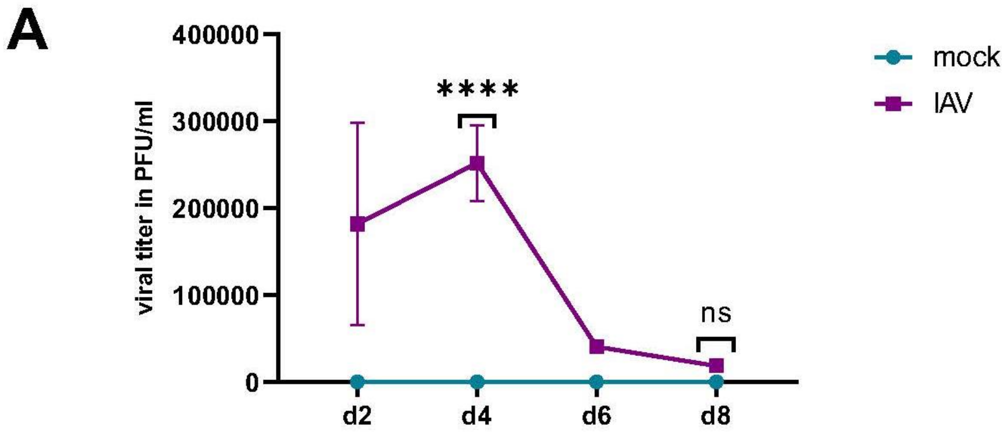

Supplementary Figure 2. (A) Ex vivo lung slices were infected with IAV for 8 d. Active virus particles determined with standard plaque assay were increased on day 4 (N = 4). Significance was calculated with Two-way ANOVA (ns $p > 0.05$; **** $p \leq 0.0001$).

© 2023. Schulz L et al. Published online at https://www.aginganddisease.org/EN/10.14336/AD.2023.0310

SUPPLEMENTARY DATA

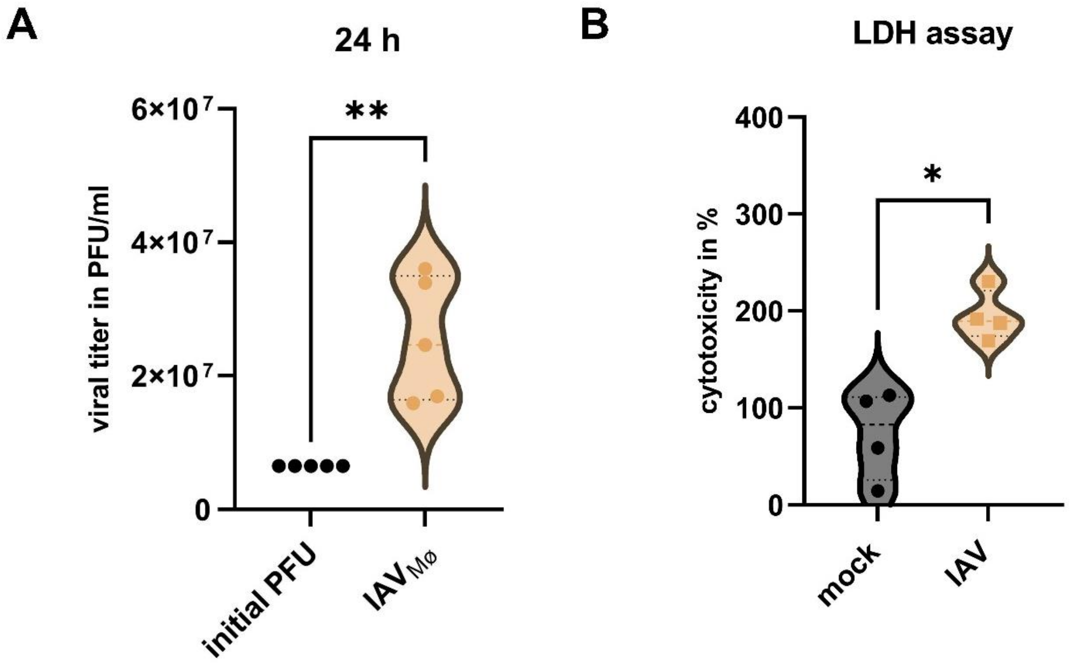

Supplementary Figure 3. (A) HMDM were infected with IAV for 24 h. Viral titer detected by standard plaque assay is significantly higher compared with the initial PFU (N = 5). Significance was calculated with the Mann-Whitney U test (** $p \leq 0.01$). (B) The infected hMDM showed significantly higher cell cytotoxicity (N = 4). Significance was calculated with the Mann-Whitney U test (* $p \leq 0.05$).

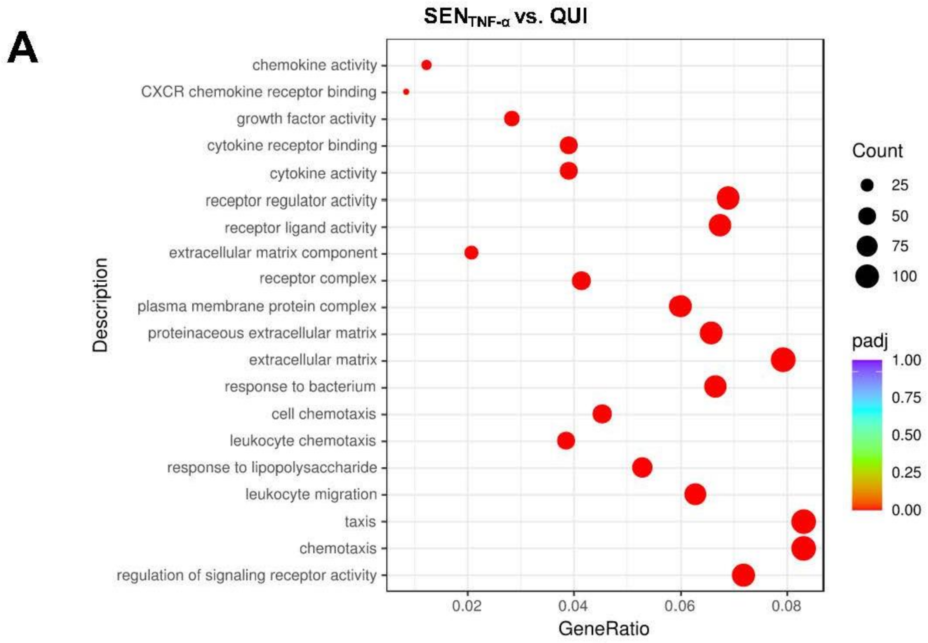

Supplementary Figure 4. (A) IMR-90s were treated with TNF-α for 10 d. The GO enrichment analysis of $SEN_{TNF\text{-}\alpha}$ in comparison to QUI demonstrated significant differences regarding extracellular matrix, chemotaxis, and regulation of signaling receptor activity.

© 2023. Schulz L et al. Published online at https://www.aginganddisease.org/EN/10.14336/AD.2023.0310

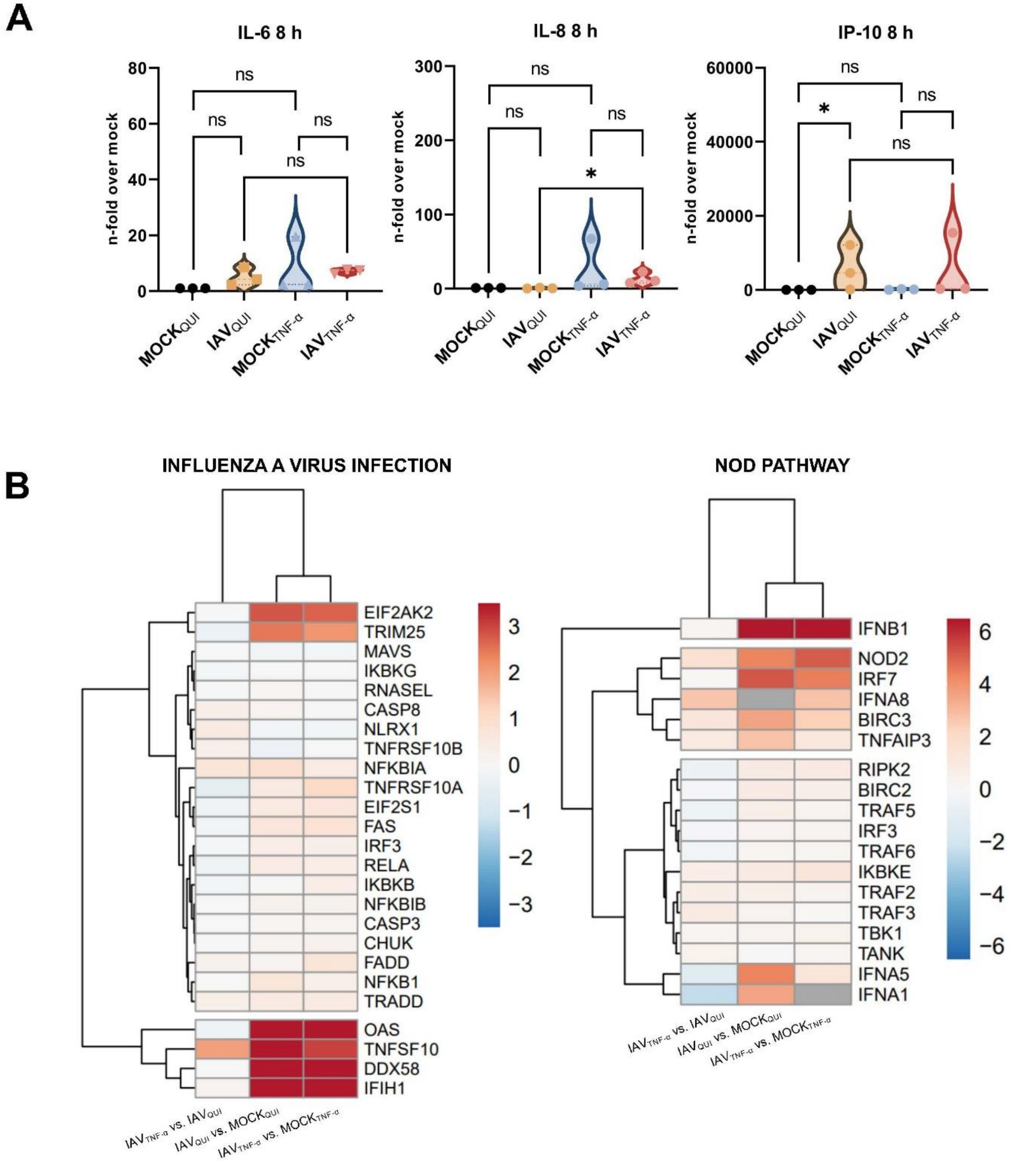

Supplementary Figure 5. (A) Senescent IMR-90s (IAV$_{TNF\text{-}\alpha}$) were infected with IAV for 8 h and 24 h. The mRNA level of IL-8 was significantly higher in IAV$_{TNF\text{-}\alpha}$ compared to IAV$_{QUI}$ (N = 3). Significance was calculated with One-way ANOVA (ns $p > 0.05$; * $p \leq 0.05$). (B) Transcriptomic analysis of IAV$_{TNF\text{-}\alpha}$ and IAV$_{QUI}$ after IAV infection. Individually expressed genes of influenza A virus infection and the NOD pathway are demonstrated in heatmaps. Gene expression in heatmaps over log2foldchange.

© 2023. Schulz L et al. Published online at https://www.aginganddisease.org/EN/10.14336/AD.2023.0310

SUPPLEMENTARY DATA

Supplementary Table 1. Transcriptomic analysis of $SEN_{TNF-\alpha}$ and SEN_{DOXO} after 10 d of senescence induction. The Venn diagram (Figure 4 F) revealed 222 co-regulated genes between $SEN_{TNF-\alpha}$ and SEN_{DOXO}.

GENE	BIOTYPE	DESCRIPTION
**GPR157**	protein coding	G protein-coupled receptor 157
**SNORA73B**	snoRNA	small nucleolar RNA, H/ACA box 73B
**ZFP69B**	protein coding	ZFP69 zinc finger protein B
**TCTEX1D4**	protein coding	Tctex1 domain containing 4
**LEPR**	protein coding	leptin receptor
**GNG12-AS1**	antisense	GNG12 antisense RNA 1
**MIR186**	miRNA	microRNA 186
**LRRC8C**	protein coding	leucine rich repeat containing 8 VRAC subunit C
**BTBD8**	protein coding	BTB domain containing 8
**ST7L**	protein coding	suppression of tumorigenicity 7 like
**MIR5087**	miRNA	microRNA 5087
**HIST2H2BC**	transcribed unprocessed pseudogene	histone cluster 2 H2B family member c (pseudogene)
**MPZ**	protein coding	myelin protein zero
**TGFB2-AS1**	antisense	TGFB2 antisense RNA 1 (head to head)
**AL118511.1**	antisense	uncharacterized LOC101927604
**B3GALNT2**	protein coding	beta-1,3-N-acetylgalactosaminyltransferase 2
**KCNS3**	protein coding	potassium voltage-gated channel modifier subfamily S member 3
**RMDN2**	protein coding	regulator of microtubule dynamics 2
**AC016727.1**	antisense	novel transcript, antisense to XPO1
**AC108479.1**	processed pseudogene	WW domain binding protein 1 (WBP1) pseudogene
**NPAS2**	protein coding	neuronal PAS domain protein 2
**AC017074.1**	lincRNA	novel transcript
**MBD5**	protein coding	methyl-CpG binding domain protein 5
**ZNF804A**	protein coding	zinc finger protein 804A
**AOX1**	protein coding	aldehyde oxidase 1
**PLEKHM3**	protein coding	pleckstrin homology domain containing M3
**ACKR3**	protein coding	atypical chemokine receptor 3
**MIR4442**	miRNA	microRNA 4442
**AC098614.1**	transcribed processed pseudogene	tropomyosin 4 (TPM4) pseudogene
**SUSD5**	protein coding	sushi domain containing 5

© 2023. Schulz L et al. Published online at https://www.aginganddisease.org/EN/10.14336/AD.2023.0310

**LINC00960**	lincRNA	long intergenic non-protein coding RNA 960
**CEP97**	protein coding	centrosomal protein 97
**QTRT2**	protein coding	queuine tRNA-ribosyltransferase accessory subunit 2
**RAB43**	protein coding	RAB43, member RAS oncogene family
**NCEH1**	protein coding	neutral cholesterol ester hydrolase 1
**LPP-AS2**	antisense	LPP antisense RNA 2
**NRROS**	protein coding	negative regulator of reactive oxygen species
**LMLN**	protein coding	leishmanolysin like peptidase
**RELL1**	protein coding	RELT like 1
**GABRA2**	protein coding	gamma-aminobutyric acid type A receptor alpha2 subunit
**LNX1**	protein coding	ligand of numb-protein X 1
**CEP135**	protein coding	centrosomal protein 135
**ADGRL3**	protein coding	adhesion G protein-coupled receptor L3
**CXCL10**	protein coding	C-X-C motif chemokine ligand 10
**BMP2K**	protein coding	BMP2 inducible kinase
**PAQR3**	protein coding	progestin and adipoQ receptor family member 3
**PRKG2**	protein coding	protein kinase cGMP-dependent 2
**HERC6**	protein coding	HECT and RLD domain containing E3 ubiquitin protein ligase family member 6
**TIFA**	protein coding	TRAF interacting protein with forkhead associated domain
**HSPA4L**	protein coding	heat shock protein family A (Hsp70) member 4 like
**RPL7AP28**	processed pseudogene	ribosomal protein L7a pseudogene 28
**NOCT**	protein coding	nocturnin
**AC093909.1**	processed pseudogene	mitochondrial translational release factor 1-like (MTRF1L) pseudogene
**MIR4458HG**	lincRNA	MIR4458 host gene
**DNAH5**	protein coding	dynein axonemal heavy chain 5
**RBBP4P1**	processed pseudogene	RB binding protein 4 pseudogene 1
**GDNF**	protein coding	glial cell derived neurotrophic factor
**GTF2H2B**	transcribed unprocessed pseudogene	general transcription factor IIH subunit 2B (pseudogene)
**ARHGEF28**	protein coding	Rho guanine nucleotide exchange factor 28
**DTWD2**	protein coding	DTW domain containing 2
**RF02038**	miscellaneous RNA	
**MFAP3**	protein coding	microfibril associated protein 3
**CYFIP2**	protein coding	cytoplasmic FMR1 interacting protein 2

© 2023. Schulz L et al. Published online at https://www.aginganddisease.org/EN/10.14336/AD.2023.0310

SUPPLEMENTARY DATA

**AC008429.2**	processed pseudogene	pyrophosphatase (inorganic) 1 (PPA1) pseudogene
**EDN1**	protein coding	endothelin 1
**HIST1H2AI**	protein coding	histone cluster 1 H2A family member i
**ZSCAN12P1**	transcribed unprocessed pseudogene	zinc finger and SCAN domain containing 12 pseudogene 1
**UHRF1BP1**	protein coding	UHRF1 binding protein 1
**USP45**	protein coding	ubiquitin specific peptidase 45
**PRDM1**	protein coding	PR/SET domain 1
**MFSD4B**	protein coding	major facilitator superfamily domain containing 4B
**RNA5SP219**	rRNA pseudogene	RNA, 5S ribosomal pseudogene 219
**ULBP2**	protein coding	UL16 binding protein 2
**ULBP1**	protein coding	UL16 binding protein 1
**ULBP3**	protein coding	UL16 binding protein 3
**IL6**	protein coding	interleukin 6
**GTF2IP23**	unprocessed pseudogene	general transcription factor IIi pseudogene 23
**MIR590**	miRNA	microRNA 590
**RWDD4P1**	processed pseudogene	RWD domain containing 4 pseudogene 1
**ATXN7L1**	protein coding	ataxin 7 like 1
**WNT16**	protein coding	Wnt family member 16
**AC006148.2**	lincRNA	novel transcript
**AC004930.1**	lincRNA	novel transcript
**BX890604.2**	lincRNA	novel transcript
**MIR4767**	miRNA	microRNA 4767
**TBCAP1**	processed pseudogene	tubulin folding cofactor A pseudogene 1
**MID1IP1-AS1**	antisense	MID1IP1 antisense RNA 1
**CLCN5**	protein coding	chloride voltage-gated channel 5
**MIR6895**	miRNA	microRNA 6895
**PHKA1**	protein coding	phosphorylase kinase regulatory subunit alpha 1
**RBM41**	protein coding	RNA binding motif protein 41
**L1CAM**	protein coding	L1 cell adhesion molecule
**TMLHE**	protein coding	trimethyllysine hydroxylase, epsilon
**NAT1**	protein coding	N-acetyltransferase 1
**TNFRSF10C**	protein coding	TNF receptor superfamily member 10c
**TNFRSF10A**	protein coding	TNF receptor superfamily member 10a

© 2023. Schulz L et al. Published online at https://www.aginganddisease.org/EN/10.14336/AD.2023.0310

**DOCK5**	protein coding	dedicator of cytokinesis 5
**AC108449.2**	sense intronic	novel transcript, sense intronic to HMBOX1
**DUSP4**	protein coding	dual specificity phosphatase 4
**RAB11FIP1**	protein coding	RAB11 family interacting protein 1
**POMK**	protein coding	protein-O-mannose kinase
**MYBL1**	protein coding	MYB proto-oncogene like 1
**AC087752.3**	sense intronic	novel transcript
**C8orf31**	processed transcript	chromosome 8 open reading frame 31 (putative)
**GLIS3**	protein coding	GLIS family zinc finger 3
**PRUNE2**	protein coding	prune homolog 2
**TNFSF15**	protein coding	TNF superfamily member 15
**ASTN2**	protein coding	astrotactin 2
**AC080023.1**	antisense	novel transcript
**SOX6**	protein coding	SRY-box 6
**NCR3LG1**	protein coding	natural killer cell cytotoxicity receptor 3 ligand 1
**DNAJC24**	protein coding	DnaJ heat shock protein family (Hsp40) member C24
**AC087276.1**	sense overlapping	novel transcript, sense overlapping TTC17
**PTPRJ**	protein coding	protein tyrosine phosphatase, receptor type J
**BATF2**	protein coding	basic leucine zipper ATF-like transcription factor 2
**FAM86C1**	protein coding	family with sequence similarity 86 member C1
**ALG1L9P**	transcribed unprocessed pseudogene	asparagine-linked glycosylation 1-like 9, pseudogene
**RNF169**	protein coding	ring finger protein 169
**AP003119.2**	antisense	uncharacterized LOC101928837
**ACER3**	protein coding	alkaline ceramidase 3
**CCDC89**	protein coding	coiled-coil domain containing 89
**MMP10**	protein coding	matrix metallopeptidase 10
**ZC3H12C**	protein coding	zinc finger CCCH-type containing 12C
**USP6NL**	protein coding	USP6 N-terminal like
**NAMPTP1**	processed pseudogene	nicotinamide phosphoribosyltransferase pseudogene 1
**PANK1**	protein coding	pantothenate kinase 1
**EXOC6**	protein coding	exocyst complex component 6
**SLC35G1**	protein coding	solute carrier family 35 member G1
**PLEKHA1**	protein coding	pleckstrin homology domain containing A1
**ADAM12**	protein coding	ADAM metallopeptidase domain 12

© 2023. Schulz L et al. Published online at https://www.aginganddisease.org/EN/10.14336/AD.2023.0310

**AC010186.3**	sense intronic	novel transcript
**EIF2S3B**	protein coding	eukaryotic translation initiation factor 2 subunit gamma B
**FAM234B**	protein coding	family with sequence similarity 234 member B
**BHLHE41**	protein coding	basic helix-loop-helix family member e41
**FAR2**	protein coding	fatty acyl-CoA reductase 2
**SLC2A13**	protein coding	solute carrier family 2 member 13
**AMIGO2**	protein coding	adhesion molecule with Ig like domain 2]
**SCN8A**	protein coding	sodium voltage-gated channel alpha subunit 8
**TAC3**	protein coding	tachykinin 3
**RXYLT1**	protein coding	ribitol xylosyltransferase 1
**PABPC1P4**	processed pseudogene	poly(A) binding protein cytoplasmic 1 pseudogene 4
**RBMS1P1**	processed pseudogene	RNA binding motif single stranded interacting protein 1 pseudogene 1
**SLC35E3**	protein coding	solute carrier family 35 member E3
**LINC02407**	lincRNA	long intergenic non-protein coding RNA 2407
**NAV3**	protein coding	neuron navigator 3
**GIT2**	protein coding	GIT ArfGAP 2
**P2RX7**	protein coding	purinergic receptor P2X 7
**MPHOSPH9**	protein coding	M-phase phosphoprotein 9
**AL161421.1**	antisense	novel transcript, antisense to FNDC3A
**KLF5**	protein coding	Kruppel like factor 5
**SNORA79B**	snoRNA	small nucleolar RNA, H/ACA box 79B
**ARHGAP5**	protein coding	Rho GTPase activating protein 5
**RALGAPA1**	protein coding	Ral GTPase activating protein catalytic alpha subunit 1
**KLHL28**	protein coding	kelch like family member 28
**HIF1A-AS1**	lincRNA	HIF1A antisense RNA 1
**COQ6**	protein coding	coenzyme Q6, monooxygenase
**ENTPD5**	protein coding	ectonucleoside triphosphate diphosphohydrolase 5
**AL355102.5**	antisense	novel transcript, antisense to BDKRB2
**MIR377**	miRNA	microRNA 377
**NIPA1**	protein coding	NIPA magnesium transporter 1
**PWAR5**	TEC	Prader Willi/Angelman region RNA 5
**AC124312.2**	lincRNA	novel transcript
**TTBK2**	protein coding	tau tubulin kinase 2
**TMEM62**	protein coding	transmembrane protein 62
**DMXL2**	protein coding	Dmx like 2

© 2023. Schulz L et al. Published online at https://www.aginganddisease.org/EN/10.14336/AD.2023.0310

SUPPLEMENTARY DATA

**PAQR5**	protein coding	progestin and adipoQ receptor family member 5
**SYNM**	protein coding	synemin
**MIR5587**	miRNA	microRNA 5587
**AC141586.1**	transcribed unprocessed pseudogene	potassium channel tetramerization domain containing 5 pseudogene
**ZNF597**	protein coding	zinc finger protein 597
**AC138969.1**	unprocessed pseudogene	polycystic kidney disease 1 (autosomal dominant) (PKD1) pseudogene
**CCP110**	protein coding	centriolar coiled-coil protein 110
**RAB43P1**	processed pseudogene	RAB43 pseudogene 1
**ARL2BP**	protein coding	ADP ribosylation factor like GTPase 2 binding protein
**CMTM1**	protein coding	CKLF like MARVEL transmembrane domain containing 1
**NFATC3**	protein coding	nuclear factor of activated T cells 3
**SLC7A6**	protein coding	solute carrier family 7 member 6
**TANGO6**	protein coding	transport and golgi organization 6 homolog
**NUDT7**	protein coding	nudix hydrolase 7
**CENPN**	protein coding	centromere protein N
**LINC01081**	lincRNA	long intergenic non-protein coding RNA 1081
**PITPNA-AS1**	antisense	PITPNA antisense RNA 1
**AC130689.1**	processed pseudogene	succinate dehydrogenase complex, subunit C, integral membrane protein, 15kDa (SDHC) pseudogene
**ZNF594**	protein coding	zinc finger protein 594
**UBE2SP1**	processed pseudogene	ubiquitin conjugating enzyme E2 S pseudogene 1
**RASD1**	protein coding	ras related dexamethasone induced 1
**TWF1P1**	processed pseudogene	twinfilin 1 pseudogene 1
**AC060780.2**	processed pseudogene	ribosomal protein, large, P1 (RPLP1) pseudogene
**TMEM106A**	protein coding	transmembrane protein 106A
**AC004596.1**	antisense	novel transcript, antisense to ATXN7L3
**AC068152.1**	processed transcript	novel transcript
**ERN1**	protein coding	endoplasmic reticulum to nucleus signaling 1
**PLEKHM1P1**	transcribed unprocessed pseudogene	pleckstrin homology and RUN domain containing M1 pseudogene 1
**SLC16A6**	protein coding	solute carrier family 16 member 6
**AC132938.3**	antisense	novel transcript, antisense NARF
**TYMSOS**	bidirectional promoter lncRNA	TYMS opposite strand
**AC090772.3**	lincRNA	novel transcript
**KCTD1**	protein coding	potassium channel tetramerization domain containing 1

© 2023. Schulz L et al. Published online at https://www.aginganddisease.org/EN/10.14336/AD.2023.0310

**GAREM1**	protein coding	GRB2 associated regulator of MAPK1 subtype 1
**RAB27B**	protein coding	RAB27B, member RAS oncogene family
**WDR7**	protein coding	WD repeat domain 7
**AC090409.1**	lincRNA	novel transcript
**RNF152**	protein coding	ring finger protein 152
**PIGN**	protein coding	phosphatidylinositol glycan anchor biosynthesis class N
**SRXN1**	protein coding	sulfiredoxin 1
**UBE2D3P1**	processed pseudogene	ubiquitin conjugating enzyme E2 D3 pseudogene 1
**MCM8**	protein coding	minichromosome maintenance 8 homologous recombination repair factor
**APCDD1L**	protein coding	APC down-regulated 1 like
**ZSWIM4**	protein coding	zinc finger SWIM-type containing 4
**MIR641**	miRNA	microRNA 641
**ZNF283**	protein coding	zinc finger protein 283
**ZNF155**	protein coding	zinc finger protein 155
**ZNF611**	protein coding	zinc finger protein 611
**ZNF702P**	transcribed processed pseudogene	zinc finger protein 702, pseudogene
**AC008735.4**	antisense	novel transcript, antisense to ZNF865
**ZNF543**	protein coding	zinc finger protein 543
**ZNRF3**	protein coding	zinc and ring finger 3
**OSBP2**	protein coding	oxysterol binding protein 2
**CCDC134**	protein coding	coiled-coil domain containing 134
**FP671120.4**	lincRNA	novel transcript, similar to YY1 associated myogenesis RNA 1 YAM1
**MEMO1P1**	processed pseudogene	mediator of cell motility 1 pseudogene 1
**TRAPPC10**	protein coding	trafficking protein particle complex 10

© 2023. Schulz L et al. Published online at https://www.aginganddisease.org/EN/10.14336/AD.2023.0310

SUPPLEMENTARY DATA

Supplementary Table 2. Listed are the experimental models, primers, antibodies, chemicals, proteins, kits, softwares and advices utilized in this paper

EXPERIMENTAL MODEL	DESCRIPTION	COMPANY	CATALOG
***in vitro* primary cell**	IMR-90 – normal human fetal lung fibroblast	Coriell Institute for Medical Research	I90-83
***in vitro* cell line**	Madin-Darby canine kidney (MDCK)	Institute of Medical Microbiology, Jena	N/A
***in vivo* model**	BALB/cJRj-wild-type mice	Janvier Labs	N/A
***ex vivo* model**	C57/bl6-wild type mice	University Hospital Jena	N/A
***in vitro* virus strain**	influenza A virus/IAV/H1N1/Puerto Rico/8/1934	Institute of Medical Microbiology, Jena	N/A
***in vivo* virus strain**	influenza A virus/IAV/H1N1/vi0084/2016	Institute of Medical Microbiology, Jena	N/A
***ex vivo* virus strain**	HA-G222-mpJena/5258	Institute of Medical Microbiology, Jena	N/A

PRIMER	SEQUENCE FORWARD	SEQUENCE REVERSE
**β-Actin mouse**	ATGGAGGGGAATACAGCCC	TTCTTTGCAGCTCCTTCGTT
**CDKN2A mouse**	AATCTCCGCGAGGAAAGC	GTCTGCAGCGGACTCCAT
**CDKN1A mouse**	TTGCCAGCAGAATAAAAGGTG	TTTGCTCCTGTGCGGAAC
**MMP3 mouse**	CGATGGACAGAGGATGTCAC	CAGCCTTGGCTGAGTGGT
**Lamin B1 mouse**	GGGAAGTTTATTTCGCTTGAAGA	ATCTCCCAGCCTCCCATT
**β-Actin human**	CATGTACGTTGCTATCCAGGC	CTCCTTAATGTCACGCACGAT
**CDKN2A human**	GAGCAGCATGGAGCCTTC	CGTAACTATTCGGTGCGTTG
**CDKN1A human**	TCACTGTCTTGTACCCTTGTGC	GGCGTTTGGAGTGGTAGAAA
**MMP3 human**	CAAAACATATTTCTTGTAGAGGACAA	TTCAGCTATTTGCTTGGGAAA
**Lamin B1 human**	TTGGATGCTCTTGGGGTTC	AAGCAGCTGGAGTGGTTGTT
**IL-6 human**	AATTCGGTACATCCTCGACGG	GGTTGTTTTCTGCCAGTGCC
**IP-10 human**	CCAGAATCGAAGGCCATCAA	TTTCCTTGCTAACTGCTTTCAG
**IL-8 human**	ATGACTTCCAAGCTGGCCGTGGCT	TCTCAGCCCTCTTCAAAAACTTCT

ANTIBODY	DESCRIPTION	COMPANY	CATALOG
**p16**	p16INK4a Polyclonal Antibody	Thermo Fisher Scientific	PA5-20379
**p21**	p21 (WAF1, Cip1) Polyclonal Antibody	Thermo Fisher Scientific	14-6715-81
**DPP4**	CD26 Recombinant Rabbit Monoclonal Antibody (JM11-42)	Thermo Fisher Scientific	MA5-32643
**phalloidin**	BODIPY™ 558/568 Phalloidin	Thermo Fisher Scientific	B3475
**secondary p16, p21, DPP4**	Alexa Fluor® 488 AffiniPure Donkey Anti-Rabbit IgG (H+L)	Jackson Immuno Research	711-545-152
**isotype control**	Rabbit (DA1E) mAb IgG XP® Isotype Control	Cell Signaling	3900
**IAV nucleoprotein**	Anti-H1N1 Influenza A virus Nucleocapsid protein antibody	Abcam	ab104870
**CD68**	CD68 Monoclonal Antibody (KP1)	Thermo Fisher Scientific	14-0688-82
**secondary IAV nucleoprotein**	Alexa Fluor® 488 AffiniPure Donkey Anti-Rabbit IgG (H+L)	Jackson Immuno Research	711-545-152
**secondary CD68**	Cy™3 AffiniPure Donkey Anti-Mouse IgG (H+L)	Jackson Immuno Research	715-165-150
**DPP4**	DPP4/CD26 (D6D8K) Rabbit mAb	Cell Signaling	67138S
**p21**	p21 Waf1/Cip1 (12D1) Rabbit mAb	Cell Signaling	2947S
**ß-Actin**	Monoclonal Anti-ß-Actin antibody produced in mouse	Sigma Aldrich	A2228-100UL
**HRP DPP4, p21**	Goat Anti-Rabbit IgG (H+L)-HRP Conjugate	Biorad	172-1019
**HRP ß-Actin**	Goat Anti-Mouse IgG (H+L)-HRP Conjugate	Biorad	172-1011
**IAV nucleoprotein**	Anti-H1N1 Influenza A virus Nucleocapsid protein antibody	Abcam	ab104870

© 2023. Schulz L et al. Published online at https://www.aginganddisease.org/EN/10.14336/AD.2023.0310

SUPPLEMENTARY DATA

**phalloidin**	Alexa Fluor™ Plus 647 Phalloidin	Thermo Fisher Scientific	A30107
**secondary IAV nucleoprotein**	Cy3-conjugated AffiniPure Donkey Anti-Rabbit IgG (H+L)	Jackson Immuno Research	711-165-152
**nuclei**	DAPI Fluoromount-G®	Southern Biotech	0100-20

CHEMICAL, PROTEIN	DESCRIPTION	COMPANY	CATALOG
**10x MEM**	MEM (10X) Minimum Essential Medium	Thermo Fisher Scientific	21430020
**Agar**	Oxoid Purified Agar	Thermo Fisher Scientific	LP0028B
**Agarose**	Agarose (4 %)	Thermo Fisher Scientific	18300012
**Albumin**	Albumine solution 30 %	Roth	9401.3
**$CaCl_2$**	Calcium chloride	Sigma Aldrich	746495-100G
**DAPI**	DAPI Fluoromount-G®	Southern Biotech	0100-20
**Dextran**	DEAE Dextran	Roth	4198.2
**DMEM**	Dulbecco's Modified Eagle's Medium	Sigma Aldrich	D6429
**DMEM/F-12**	DMEM/F-12, no phenol red	Thermo Fisher Scientific	21041025
**DMEM/F-12 GlutaMAX**	DMEM/F-12, GlutaMAX™ Supplement	Thermo Fisher Scientific	31331093
**Doxorubicin**	Doxorubicin hydrochloride	TOCRIS	2252
**DPBS**	DPBS, no calcium, no magnesium	Thermo Fisher Scientific	14190-169
**EMEM**	Minimum Essential Medium Eagle	Sigma Aldrich	M4655-500ML
**FBS**	FBS standard, South America origin, fetal bovene serum	Pan Biotech	P30-3306
**Formaldehyd**	Formaldehyd solution	Sigma Aldrich	252549-25ML
**HEPES**	HEPES (1M)	Thermo Fisher Scientific	15630056
**Histopaque**	Histopaque®-1077	Sigma Aldrich	10771-500ML
**HRP substrate**	Immobilon Western HRP-Substrate	Merck Millipore	WBKLS0500
**HSA**	Human Serum Albumin (HSA), 20 % Solution, Premium Grade	Pan Biotech	P06-27500
**M1-Macrophage Generation Medium**	M1-Macrophage Generation Medium DXF	PromoCell	C-28055
**$MgCl_2$**	Magnesium chloride	Sigma Aldrich	8147330100
**Monocyte Attachment Medium**	Monocyte Attachment Medium	PromoCell	C-28051
**Neutral red**	Neutral red staining	Sigma Aldrich	N4638-5G
**Penicillin/Streptomycin**	Penicillin-Streptomycin Mixture	Lonza	DE17-602E
**PVDF**	PVDF Transfer Membrane	Thermo Fisher Scientific	88518
**RIPA buffer**	RIPA-Lyse- und Extraktionspuffer	Thermo Fisher Scientific	89900
**RPMI**	RPMI 1640 Medium, GlutaMAX™ Supplement	Thermo Fisher Scientific	61870044
**TNF-alpha human**	Human TNF-alpha Recombinant Protein	Thermo Fisher Scientific	PHC3011
**TNF-alpha murine**	Recombinant murine Tumor Necrosis Factor-alpha (rm TNF-alpha)	ImmunoTools	12343016
**Triton X**	Triton X 100	Roth	3051.2
**Trypsin**	Trypsin, TPCK Treated	Thermo Fisher Scientific	20233

KIT	COMPANY	CATALOG
**CyQUANT™ LDH Cytotoxicity Assay**	Thermo Fisher Scientific	C20300
**High-Capacity cDNA Reverse Transcription Kit**	Thermo Fisher Scientific	4368813
**LEGENDplex™ Human Inflammation Panel 1 (13-plex) with Filter Plate**	Biolegend	740808
**LEGENDplex™ Human Macrophage/Microglia Panel (13-plex) with Filter Plate**	Biolegend	740502
**LEGENDplex™ Mouse Anti-Virus Response Panel (13plex) with Filter Plate**	Biolegend	740621
**Maxima SYBR Green qPCR Master Mix (2X), with separate ROX vial**	Thermo Fisher Scientific	K0253
**Pierce™ BCA Protein Assay Kit**	Thermo Fisher Scientific	23227
**RNeasy Mini QIAcube Kit (240)**	Qiagen	74116
**RNeasy Plus Micro Kit**	Qiagen	74034
**Senescence Detection Kit**	Biovision	K320

SOFTWARE	COMPANY	SOURCE
**Biorender**	Biorender	https://biorender.com/
**GraphPad Prism Version 9**	Graphpad	https://www.graphpad.com/
**Legendplex Data Analysis Software Version 8**	biolegend	https://www.biolegend.com/

© 2023. Schulz L et al. Published online at https://www.aginganddisease.org/EN/10.14336/AD.2023.0310

SUPPLEMENTARY DATA

**R**	R Foundation for Statistical Computing	https://www.R-project.org/
**ZEN 2 (blue edition)**	Carl Zeiss	https://www.zeiss.com/

ADVICE	DESCRIPTION	COMPANY
**Accuri C6 Plus Cytometer**	flow cytometer	BD Biosciences
**AxioObserver Z.1+Apotome 2**	microscope	Zeiss
**FusionFX**	chemiluminescence imaging	Vilber
**Leica CM1950**	cryostat	Leica
**Leica VT1200 S**	vibratome	Leica
**NanoDrop spectrophotometer ND-1000**	spectrophotometer	Peqlab/VWR
**Qubit 4**	fluorometer	Thermo Fisher Scientific

© 2023. Schulz L et al. Published online at https://www.aginganddisease.org/EN/10.14336/AD.2023.0310
